# Identification of Y chromosome markers in the eastern three-lined skink (*Bassiana duperreyi*) using in silico whole genome subtraction

**DOI:** 10.1186/s12864-020-07071-2

**Published:** 2020-09-29

**Authors:** Duminda Sampath Bandara Dissanayake, Clare Ellen Holleley, Laura Kate Hill, Denis O’Meally, Janine Eileen Deakin, Arthur Georges

**Affiliations:** 1 Institute for Applied Ecology, University of Canberra, Canberra, ACT 2601 Australia; 2 Australian National Wildlife Collection, CSIRO, Canberra, ACT 2911 Australia; 3 Present Address: Centre for Gene Therapy, Beckman Research Institute of the City of Hope, Duarte, CA USA

**Keywords:** Sex-specific markers, Sex reversal, Genotypic sex determination, Y chromosome

## Abstract

**Background:**

Homologous sex chromosomes can differentiate over time because recombination is suppressed in the region of the sex determining locus, leading to the accumulation of repeats, progressive loss of genes that lack differential influence on the sexes and sequence divergence on the hemizygous homolog. Divergence in the non-recombining regions leads to the accumulation of Y or W specific sequence useful for developing sex-linked markers. Here we use in silico whole-genome subtraction to identify putative sex-linked sequences in the scincid lizard *Bassiana duperreyi* which has heteromorphic XY sex chromosomes.

**Results:**

We generated 96.7 × 10^9^ 150 bp paired-end genomic sequence reads from a XY male and 81.4 × 10^9^ paired-end reads from an XX female for in silico whole genome subtraction to yield Y enriched contigs. We identified 7 reliable markers which were validated as Y chromosome specific by polymerase chain reaction (PCR) against a panel of 20 males and 20 females.

**Conclusions:**

The sex of *B. duperreyi* can be reversed by low temperatures (XX genotype reversed to a male phenotype). We have developed sex-specific markers to identify the underlying genotypic sex and its concordance or discordance with phenotypic sex in wild populations of *B. duperreyi*. Our pipeline can be applied to isolate Y or W chromosome-specific sequences of any organism and is not restricted to sequence residing within single-copy genes. This study greatly improves our knowledge of the Y chromosome in *B. duperreyi* and will enhance future studies of reptile sex determination and sex chromosome evolution.

## Background

Most vertebrates reproduce sexually with distinct male and female phenotypes that arise from the complement of chromosomes that are inherited from their parents. These species are said to have their sex determined genotypically (GSD), and the influential genes reside on sex chromosomes that typically assort randomly during meiosis. In the absence of differential investment by the parents in male and female offspring, this system yields an evolutionarily stable 1:1 primary offspring sex ratio [1–3].

Sex chromosomes are thought to evolve from autosomes when genes they carry assume the role of determining sex [4]. What follows over time is a chain of mutational events on the hemizygous member of the sex chromosome pair, leading to the accumulation of genes that afford a fitness advantage to the heterogametic sex, a fitness disadvantage to the homogametic sex, suppression of recombination, the accumulation of repetitive sequence, and progressive loss of gene function unrelated to sex [5, 6]. In humans, for example, the non-recombining region of the Y chromosome contains 78 protein coding genes encoding 27 proteins [7] compared with the 699 protein-coding genes with known function on the X [8]; the human Y is smaller than the X and highly heterochromatic.

Unlike mammals, squamates show a remarkable diversity in sex chromosome structure, representing various degrees of differentiation in sex homologs [9–13]. Such heterogeneity is brought about by variation in the evolutionary age of lineages with independently evolved sex chromosomes [11, 14]. In many squamate species with GSD, the sex chromosomes are homomorphic and cannot be distinguished using conventional karyotyping methods such as G or C-banding [15, 16]. In others, macroscopic differences may exist, but the sex chromosomes are microchromosomes and go undetected until more sensitive techniques, such as comparative genomic hybridisation, are applied [17, 18]. Suppression of recombination along all or part of the sex chromosome length allows homologous sequences to diverge over time [19]. Differences between sex chromosome homologues can be substantial as in human and mouse [20, 21] or very slight, involving even a single nucleotide polymorphism in an influential gene, as for *Amhr2* in the pufferfish *Takifugu rubripes* [22, 23]. For these reasons, identifying the sex chromosomes and candidate sex determining genes can be challenging, particularly for organisms that lack a reference genome. Sex-linked markers provide one important avenue for the identification of sex chromosomes and sequences that may include candidate sex determining genes [24–26].

Various approaches have been used to identify sex-linked markers in non-classical model organisms. Random amplified polymorphic DNA fingerprinting (RAPD) [27–29] and amplified fragment length polymorphisms (AFLP) [30–32] are PCR-based DNA fingerprinting techniques that sample only a fraction of the whole genome. While useful, these techniques have some drawbacks such as poor reproducibility owing to mismatches between primer and template, and difficulty in developing locus-specific markers from individual fragments. Having no knowledge of the genomic context of the typically short markers can also render interpretation difficult.

With the development of next-generation sequencing technologies, new methods have been developed for screening sex linked DNA. For example, assaying for sex-specific expressed genes by RNA-seq [33] or whole genome sequencing based approaches that rely on differences in mapped read depth [34, 35]. Restriction Site-Associated DNA sequencing (RAD-seq) or double digest restriction-site associated DNA sequencing (ddRAD-seq) is increasingly common [25, 36–43] as is DArT-seq [44–46] when searching for sex-linked sequence. These RADseq and reduced representational approaches assess only a limited portion of the genome, and may miss many markers, particularly in species with small sex-specific domains or those with micro-sex chromosomes [47].

Here, we report an in silico approach to isolate sex specific markers based on sequence unique to the Y or W chromosome, analogous to genomic representational difference analysis (gRDA) [48]. Subtractive genomic approaches have been used to identify targets in various human bacterial pathogens [49–52] and identify potential tumour antigen candidates and cancer-specific genes [53–56]. Our study is the first to apply the subtraction approach for identifying the Y chromosome specific sequence in a reptile, the eastern three-lined skink (*Bassiana duperreyi*). The species has heteromorphic XY sex chromosomes [57]. Identifying sex-specific markers for this species is of particular interest because XX individuals develop as males at low temperatures [58, 59]. Quinn et al. [32] developed AFLP markers for *B. duperreyi*, however, the fragments are short and difficult to amplify reliably. Here, we use low depth whole genome sequencing of a male and a female *B. duperreyi* to apply an in silico whole genome subtraction approach, and develop new practical markers, useful in ongoing studies of this species in the laboratory and the wild.

## Results

### In silico whole genome subtraction

We generated 96.7 × 10^9^ 150 bp PE reads from the male and 81.4 × 10^9^ PE reads from the female sequencing libraries for the in silico whole genome subtraction pipeline. This equates to approximately 8x coverage of the genome estimated from the k-mer analysis. We decomposed these reads into 14,310,783,435 and 36,695,139,446 27-mers for the male and female respectively (Additional File 1: Figs S1 and S2), the difference likely arising from differences in sequence error rates between sequencing runs. To remove k-mers arising from sequence errors, we examined the k-mer spectrum to determine suitable thresholds and eliminated k-mers with counts less than 2 for males and 5 for females to yield 1,431,111,978 and 1,483,106,252 respectively. A total of 1,129,675,305 k-mers were common to both sexes and 301,436,673 k-mers were unique to the male individual. The male-specific k-mers were reassembled to yield 15,280,950 contigs ranging from 80 bp to 1374 bp (Additional File 1: Fig. S3). Genome sizes of closely related species are between 1.9 and 2.5 GB.

### Verification of phenotypic sex identification

Three karyotyped animals whose sex was identified by hemipenal eversion and presence or absence of breeding coloration had their gonadal sex confirmed by histology and their chromosomal sex confirmed by cytology (Additional File 1: Figs S6 and S7).

### PCR validation

**Fig. 1:**
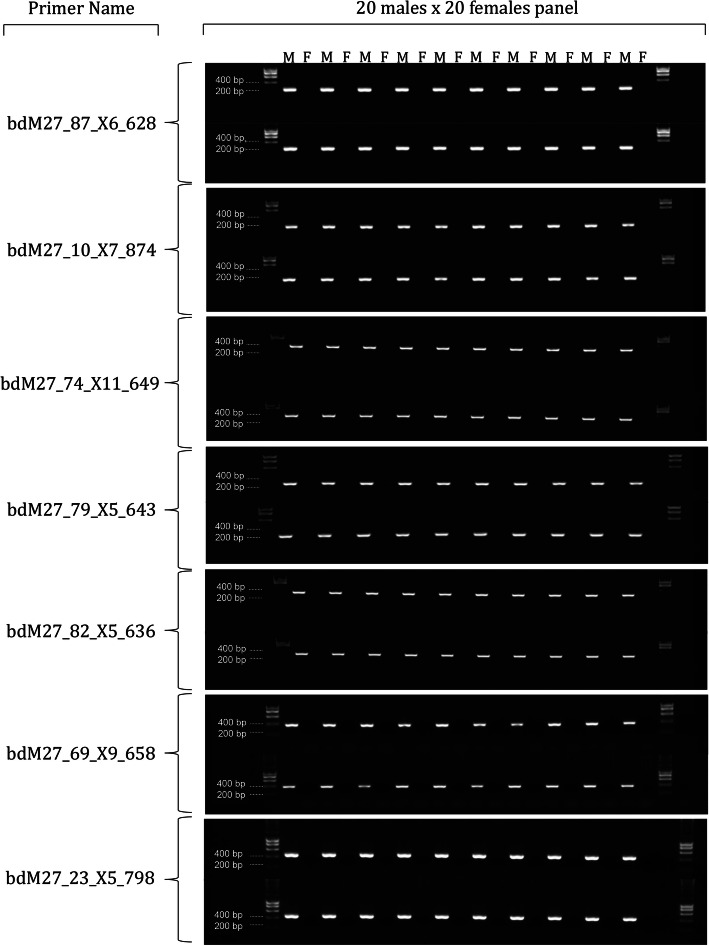
Validation of seven male-specific markers in *Bassiana duperreyi* using a panel of 20 male and 20 female individuals of confirmed phenotypic sex. Male specificity was defined as the presence of a distinct amplicon in males and the absence of amplification in females. Raw images are provided in Additional File 2

We selected the longest 92 contigs from the subtraction for further investigation, because they were of sufficient size to design robust primers and result in a PCR product easily visualised on an agarose gel. The 92 contigs ranged from 623 to 1374 bases in length (Additional File 1: Figs S4 and S5). As expected, all 92 contigs passed the subtraction validation test where a product of the expected size successfully amplified in the focal male and did not amplify in the focal female. Of these, 52 contigs yielded putative Y-chromosome markers when screened against the panel of 4 male and 4 female individuals, however, only 7 of these putative markers (Table 1) ranging in length from 628 bp to 824 bp, were validated as sex-specific when tested in the full panel of an additional 20 males and 20 females (Fig. 1). We applied the seven Y-chromosome markers to an additional 20 Anglesea animals (10 males and 10 females) and, in each case, the phenotypic sex was concordant with the genotypic sex inferred by the PCR test. Thus the 7 makers were completely concordant with phenotypic sex (present in male absent in female) in a total of 70 animals.

**Table 1:** Primers for the amplification of putative Y chromosome markers for *Bassiana duperreyi*

Primer Name	Sequence (5′–3′)		Product size (bp)
	Forward	Reverse	
bdM27_87_X6_628	TCTGAGGACATTGCAGGAACAA	GGCCTAATGAGACCTAGCAGTC	269
bdM27_10_X7_874	AAGATGGGAACTGCACTGGTAG	CAATATCCCCTGATGCAGCTCT	418
bdM27_74_X11_649	GAGGTCTGACAGAACCCTCTTG	TTTTGGTCCTGGAACAAGGTGA	286
bdM27_79_X5_643	TGTGAGACAATAGTGACCAGGC	TGCTCAGGTCTAGGGATGTGTA	294
bdM27_82_X5_636	TCTTTCTCTTTGCCCCAACCTT	ACTCTTGAATGTCGCAGTAGCA	380
bdM27_69_X9_658	TCAATGGACCTTGCATCATGGA	CCTTGGATTACTGCACTGACCT	390
bdM27_23_X5_798	TGTTCTCCGTACAATCACTGCA	TGACTTTTTGGCCGTGTAATGG	439

The sequenced PCR products were aligned to the relevant full-length subtraction contig for each of the seven Y loci. When Piccadilly Circus and Anglesea populations were compared, alignment results showed a small number of discrepancies in the nucleotide composition obtained from five of the seven amplicons (Additional File 1: Figs S8 to S14; Table S1). Of those that varied, sequence divergence ranged from 1.7% in the bdM27_79_X5_643 amplicon (Additional File 1: Fig. S12) to 0.3% in the bdM27_23_X5_798 amplicon (Additional File 1: Table S1). Both bdM27_74_X11_649 (Additional File 1: Fig. S10) and bdM27_87_X6_628 (Additional File 1: Fig. S14) amplicons were identical across populations.

### Gene and repeat identification

One of the seven Y-chromosome specific contigs, bdM27_23_X5_798, bears the partial sequence of an exon from the gene *UBE2H*, a member of a syntenic block conserved among jawed vertebrates [60]. No other significant hits were found among the 7 sauropsid genomes searched, nor from the non-redundant Genbank database. We expected that the Y-contigs would be enriched for repetitive DNA sequences, coupled with unique flanking regions, so we searched against Dfam [61], a database of transposable elements. Two contigs, bdM27_79_X5_643 and bdM27_69_X9_658, had partial matches to known murine Class 1 retrotransposon elements, and bdM27_82_X5_636 had a partial match to a DIRS endogenous retrovirus known from the painted turtle (Additional File 1: Tables S2 and S3).

## Discussion

This study is the first to use an in silico whole genome subtraction approach to successfully develop sex chromosome markers without generating a linkage map or a reference genome in a reptile species. We rapidly isolated seven robust Y chromosome markers using a user friendly and cost effective in silico whole genome subtraction pipeline. The Y-markers segregated with sex in both the Piccadilly Circus study population and a genetically distinct population of Anglesea *B. duperreyi* which have been isolated from each other since the Late Pliocene, about 3.5 Mya [62]. This suggests that, all populations retain the ancestral state and that our makers are likely to have broad applicability across the entire species range. That said, the amplified sex specific region revealed some divergence between the Anglesea population and the Piccadilly Circus populations, suggesting that mutations could occur in the primer sites of some populations/taxa, limiting the generality of the sex-linked markers. The identification of sex-specific sequence has important practical value in many contexts, including ecological studies [63–65], conservation of threatened or endangered species [66–69], captive breeding [70], aquaculture [71, 72], elimination of mortality as a possible explanation for sex ratio bias [32, 73] sex forensics [74] and identifying genotypic sex [32, 75, 76] or in studies of early developmental processes where sex of the developing embryo is important [77, 78].

Two approaches for identifying sex linked markers using whole genome sequencing seem appropriate, both relying on the divergence of the X and Y homologues in the region of recombination suppression. One technique, championed by Cortez, et al. [79] in exploring variation among mammalian species in the Y chromosome, and recently applied to the yellow-bellied water skink, *Eulamprus heatwolei* [80], is to examine read copy number across the genome and identify the half copy number in the XY individuals compared to the XX individuals after screening out repetitive sequence. This technique identifies regions that have been lost from the non-recombining region of the Y chromosome but, remain on the X chromosome, which can be developed as sex specific markers and validated using PCR [80]. Here we used as an alternative complementary approach, in silico whole genome subtraction to identify male-specific markers in the skink *B. duperreyi*, subsequently validated them using a PCR panel with individuals of known sex. Our technique is useful for identifying novel sequences, often repetitive elements, gained by the non-recombining region of the Y chromosome, or lost from the X chromosome. Neither of these approaches requires a reference genome, and so both are applicable to studies of organisms with no or incomplete reference genomes. Our technique does not require substantial read depth and thus avoids the associated high cost. Lower read depth can be a challenge because it reduces the efficiency of the subtraction approach by increasing the number of false positives. Indeed, this may have been a contributing factor to our 8% success rate. However, the ultimate goal was achieved, Y markers were discovered. Thus, PCR validation is effective at eliminating the false positives resulting from autosomal polymorphisms and differential coverage in the male and female.

Our technique decomposes a set of reads from the genome to yield a unique, but highly redundant, representation of the genome as overlapping k-mers. We then select the k-mers found only in the XY (or ZW) individual and reassemble the k-mers to yield Y (or W) enriched contigs that can be validated using PCR on a panel of individuals whose sex is known. In this way, we were able to isolate seven Y chromosome markers. There are several advantages to our in silico whole genome subtraction approach for identifying sex specific sequence when compared to AFLP, microsatellite or RAD-seq approaches. Specifically, our in silico subtraction method surveys the entire available genome, assuming adequate read depth, to identify sex specific differences and does not rely on a highly reduced representation of the genome as with RAD and ddRAD approaches, that may miss many putative markers. This is particularly important for species with small sex chromosomes or relatively small differences between the X and the Y (or Z and W) chromosomes. Our method is cost-effective because as demonstrated here, low coverage sequencing (~8x) for a single individual of each sex is sufficient to obtain informative and robust Y-chromosome (or W chromosome) markers.

We have shown that the gene *UBE2H* (Ubiquitin Conjugating Enzyme *E2 H*) is present on the Y chromosome in both *B. duperreyi* (this study) and the skink *E. heatwolei* [80]. This strongly suggests that the sex chromosomes of these two skinks share a homologous syntenic block and perhaps share homologous sex chromosomes. Ubiquitin-conjugating enzymes are encoded by a family of highly conserved genes involved in post-translational processes targeting abnormal or short-lived proteins for degradation [81]. Although various members of the ubiquitin conjugating enzyme family are involved in testes specific processes (e.g. testis-specific *UBC4*-testis in the rat, [82] and an ascidian, [83]) we make no suggestion that *UBE2H* plays a role in sex determination in these skinks, merely that it is a gene on the sex chromosomes.

Our study paves the way for future work that relies upon successful identification of chromosomal sex in wild populations of *B. duperreyi* subject to sex reversal [58, 75]. Isolating seven novel Y- chromosome markers increases the confidence of chromosomal sex identification in *B. duperreyi* because it reduces the risk of a recombination event being misinterpreted as evidence of sex reversal. Investigating the occurrence of temperature sex reversal will increase our understanding of sex reversal as a driver of sex-chromosome turn-over in the wild [75] and establish links between environmental extremes and reptile sex determining modes [84]. Also, our Y-chromosome markers can be used to identify the chromosomal sex of embryos and so enable developmental studies of sex determination and differentiation. For example, it is unknown whether *B. duperreyi* exhibits the asynchronous gonadal and genital development observed in other species with sex reversal [78]. In addition to identifying sex chromosome markers, this subtraction approach could be leveraged to identify anchor points in a draft assembly to locate genes on the sex chromosomes in non-model organisms, including candidates for sex determining genes. Pairing our marker-discovery approach with high quality whole-genome assemblies will accelerate our knowledge of sex chromosome evolution.

In this study, we identified a modest number of Y-chromosome markers, numbering 7 of 92 screened (8%). The success rate of future Y-marker discovery via genome subtraction could be improved by implementing efforts to reduce false positives caused by autosomal insertion/deletion polymorphisms in the focal sequenced individuals. This could be achieved through several complementary strategies: 1. subtracting multiple XX individuals from the XY focal individual/s; 2. selecting individuals for sequencing from populations with lower rates of heterozygosity (e.g. small geographically isolated populations or experimentally inbred lines); 3. sequencing siblings or related individuals. These improvements would increase the efficiency of sex chromosome sequence identification using whole genome subtraction.

## Conclusions

Here we describe an effective tool for characterising sex chromosomes in non-model organisms. Our approach targets sex-specific insertions and highly differentiated sex chromosome regions that are suitable for developing diagnostic sex-markers. This approach complements existing methods for identifying sex chromosome homologues and aids the classification of sex determination systems in a wide range of species. The ability of our method to provide insights about the evolutionary origins of sex chromosomes is demonstrated here by the discovery of a scincid Y-chromosome gene, common to species separated by *ca* 40 million years of evolution.

## Methods

### Samples

**Fig. 2:**
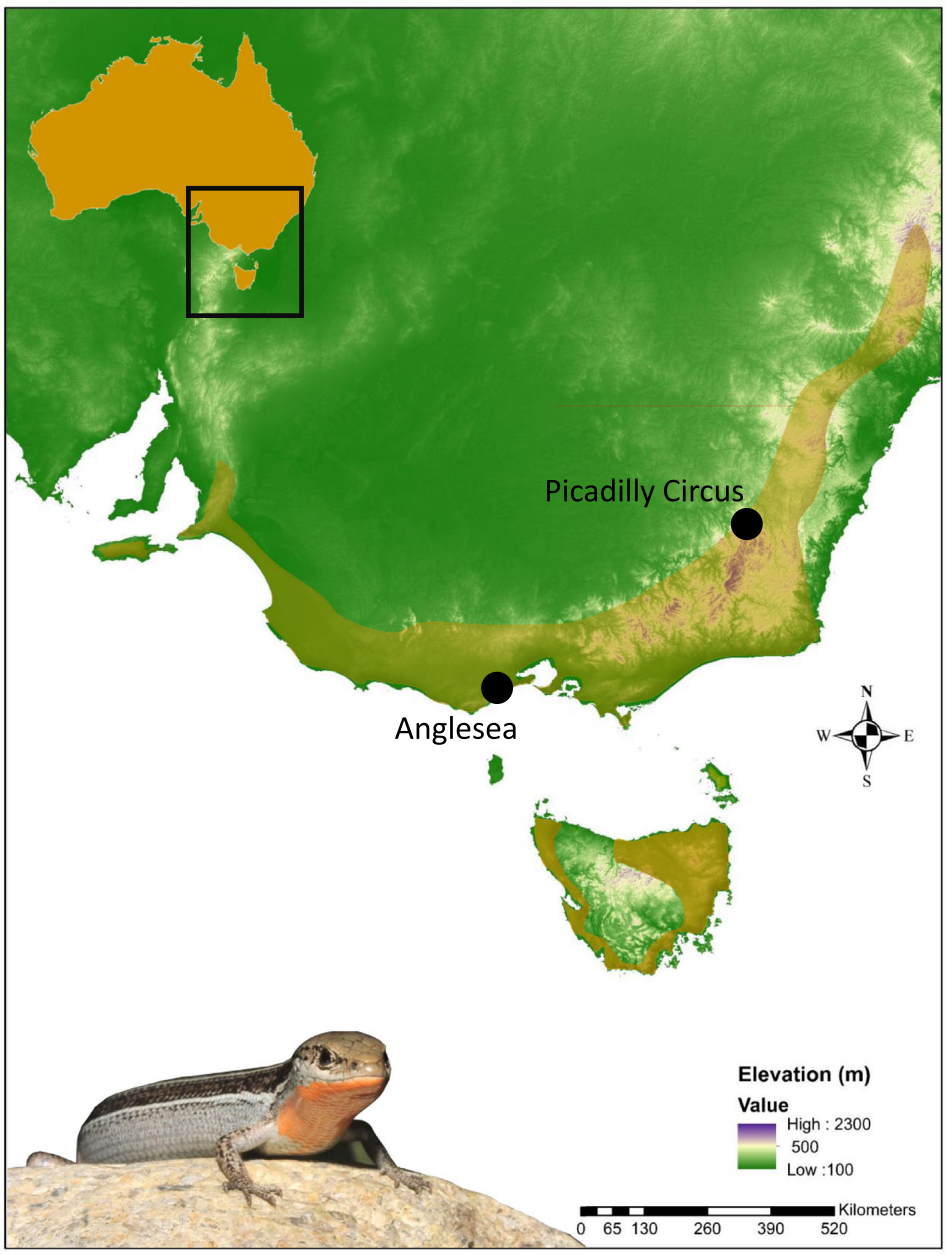
*Bassiana duperreyi* sampling localities (black circles) from which the focal and validation individuals in this study were sourced. The species approximate distribution range is indicated by the shaded area. Underlying map generated using ArcGIS 10.5.1 (http://www.esri.com) and data from the Digital Elevation Model (Geoscience Australia) made available under Creative Commons Attribution 3.0 Australia (https://creativecommons.org/licenses/by/3.0/au/legalcode, last accessed 9-Jul-20). The adult male *B. duperreyi* photo was taken by the first author at the Piccadilly Circus, ACT, Australia

The eastern three-lined skink, *B. duperreyi*, is a medium-sized (80 mm snout–vent length) lizard widely distributed through south eastern Australia, from the coast to montane cool-climate habitats [85]. Adult individuals (*n* = 76) were captured by hand at Piccadilly Circus (35°21′37.59″S, 148°48′13.39″E, 1246 m a.s.l.) in Namadgi National Park, 40 km west of Canberra in the Australian Capital Territory, and from Anglesea (38°23′26.76″S, 144°12′52.29″E, 40 m a.s.l.) in Victoria (Fig. 2, Additional File 1: Table S4). The Anglesea population is a distinct mitochondrial lineage from the Piccadilly Circus lineage (ca 3 Myr divergent, [62]). Snout-vent length was measured with Vernier callipers (+/− 1 mm) and males identified by hemipenal eversion [86] and breeding colouration. A representative male and female from Piccadilly Circus (focal individuals) were transported to the University of Canberra animal house where each was euthanised by intraperitoneal injection of sodium pentobarbitone (100–150 μg/g body weight), dissected, and phenotypic sex confirmed by examination of the gonads. Tail tips (4–5 mm) were removed with a sterile blade, a portion stored in 95% ethanol at − 20 °C, and a portion set aside for cell culture. Tail-snips were removed also from an additional 24 males and 24 females from Piccadilly Circus and 10 males and 10 females from Anglesea and stored in 95% ethanol at − 20 °C. All animals were released to the capture sites. These are referred to as the validation animals. A portion from three males and three females from Piccadilly Circus were set aside for cell culture and karyotyping.

For cell culture, tail tips were immediately transferred to 10 ml of collection medium (Gibco Dulbecco’s Modified Eagle Medium; Thermo Fisher Australia Pty Ltd., Scoresby, Victoria, Australia) with 2.5 μg/ml of Antibiotic Antimycotic Solution (Sigma Chemical Company, St. Louis, USA) and incubated at room temperature for 24 h [87] before the metaphase chromosomes preparation (see Validation of phenotypic sex identification in Methods).

### DNA extraction, sequencing, and in silico whole genome subtraction

DNA was extracted from fresh liver samples of the two focal animals and from the tail snips of the 60 validation animals using the Gentra Puregene Tissue Kit (QIAGEN, Australia) following manufacturer protocols. DNA suspensions were assessed for purity using a NanoDrop 1000 spectrophotometer (NanoDrop Technologies, Wilmington, 19,810, USA) and quantified using Qubit 2.0 fluorometer (Invitrogen, Life technologies, Sydney, NSW, Australia). Library preparation and sequencing were performed at the Biomolecular Resource Facility at the Australian National University (Canberra, ACT) using the Illumina HiSeq 2000 platform yielding 150 bp paired end reads.

**Fig. 3:**
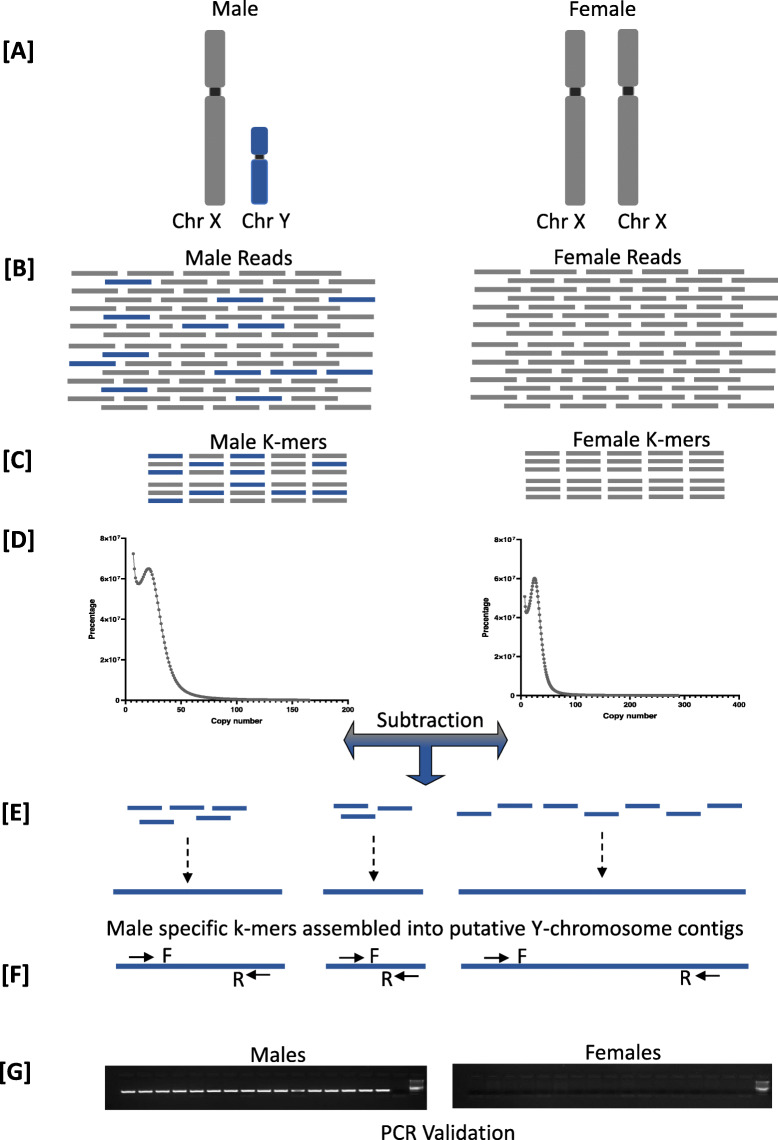
Schematic diagram showing methodology of the genome subtraction pipeline **a** A hypothetical schematic of the *B. duperreyi* sex chromosomes with the male specific gene region indicated in blue (not to scale); **b** Low coverage whole genome sequencing was conducted on an Illumina platform resulting in approximately 8X coverage; **c** The raw sequencing reads are decomposed into 27 base pair k-mers **d** The k-mer spectrum is plotted and sequences with low counts are removed; **e** Female k-mers are subtracted from the male k-mers. Male specific k-mers are retained and then assembled into putative Y-chromosome contigs; **f** Primers are designed on putative male contigs. **g** PCR sex test and validation (image shown here is for illustrative purposes only; refer to Fig. 1 and the original gel images in Additional Data 2 for the definitive data)

Reads from the focal male and the focal female were analysed independently as follows (Fig. 3). First, overlapping read pairs were combined into fragments then decomposed into k-mers of 27 bp using Jellyfish 2.0 [88]. Unique k-mers were counted, again using Jellyfish 2.0 and k-mers in common between the male and female sets were removed from the male set. This yielded a (subtracted) k-mer set that was enriched for Y chromosome sequence. Strictly, the subtracted k-mer set contains k-mers that are from Y chromosome sequence admixed with k-mers representing polymorphic differences between the female X chromosomes and the male X chromosome. K-mers in the subtraction with a count less than 2 for males and 5 for females were considered to represent sequencing errors and were removed from the analysis. This decision was based on examination of the k-mer spectra, identifying the minima immediately to the right of the peak arising from presumed read errors. This is not a critical decision. Select it too high, and the risk is that some important k-mers will be eliminated from the re-assembly of Y enriched kmers. Select it too low, and the cost is inclusion of low count kmers from reads containing errors and a greater noise to signal ratio. This does not affect the outcome, just the computational resources required for subtraction and reassembly.

The remaining Y enriched k-mers were then reassembled into contigs using an inchworm assembler (kassemble.cgi, 10.5061/dryad.pvmcvdnj1) with stringent extension criteria. Briefly, the assembler initially took a focal k-mer at random and searched for other k-mers that matched exactly k-1 bp of the focal k-mer. If this second k-mer was unique, then the focal k-mer was extended by one bp, and the process was repeated. If the k-mer was not unique, then the extension process was terminated. The extension occurred to both the left and the right, yielding relatively short contigs (up to ca 1400 bp) that contain sequence unique to the male individual.

### PCR validation

To validate the sex specificity of each of the contigs and remove false positives derived from autosomal and X chromosome polymorphisms, we designed primers for each contig using Primer 3 [89] implemented in Geneious [90] (version R8). We then applied these presence/absence PCR tests in the validation animals using the following conditions. Each reaction contained 1x My Taq HS Red mix (Bioline), 4 μM each primer and 25 ng of genomic DNA. The PCR cycling conditions used an initial touchdown phase to increase the specificity of amplification: denaturing at 95 °C, annealing temperature stepping down from 70 °C by 0.5 °C for 10 cycles, extension at 72 °C. This was followed by 30 cycles at 65 °C annealing and 72 °C extension.

The PCR screening process was conducted in three stages. To confirm that the subtraction pipeline had successfully identified a presence/absence polymorphism in the two focal individuals, we first screened those two individuals to confirm presence of an amplified fragment in the male and the absence of an amplified fragment in the female. We then screened a panel of an additional 4 male and 4 female individuals for putative sex-linked markers showing a male-specific positive pattern. In a third step, we screened those putative markers on a further 20 males and 20 females from Piccadilly Circus. At each of the stages, the loci that did not appear as sex specific were eliminated as candidate sex markers. The probability of an autosomal or X chromosome polymorphism being present in the focal male, 4 males and 20 additional males, and absent in the focal female, 4 females and 20 additional females, is sufficiently low (≤ 0.25^24^, maximal for autosomal or X allele frequency = 0.5) to eliminate false positives, despite the error rate compounding over multiple markers. Thus, male specific markers that survive the validation process are Y-specific markers.

To confirm the amplification of the desired sequence, PCR products for all 7 putative Y-loci were visually assessed using gel electrophoresis and then Sanger sequenced in a single direction, using the forward primer, on an AB 3730xl DNA Analyzer at the Biomolecular Research Facility, Australian National University, Canberra, Australia. We sequenced 4 male individuals from Piccadilly Circus (Namadgi National Park, ACT) and 4 male individuals from Anglesea (Victoria).

### Validation of phenotypic sex identification

The phenotypic sex of each of the karyotyped animals was confirmed by gross examination of gonads followed by histological examination. Dissected gonads were dehydrated through graduations of ethanol (70, 90, 100%) and two changes of xylene for 45 min each, before being embedded in paraffin wax, and sectioned 5 to 6 μm using a Leica Rotary Microtome (Leica Microsystems Pty Ltd., Waverley, Australia). Slides were stained with haematoxylin and eosin, with a staining time of 2–3 min in haematoxylin, and 10 dips in 0.25% eosin in 80% ethanol, before being mounted in Depex medium (BDH Laboratory Supplied, England). Gonads were characterized according to standard cellular structures [91, 92].

Karyotyping was carried out by examining metaphase chromosomes prepared from fibroblast cell lines of tail tissues as outlined by Ezaz et al. [10] with minor modifications. Briefly, three replicate subsamples for each individual were made using sterile scalpel blade. The individual subsamples were transferred to separate T25 culture flasks with 1.5 ml Amnio-Max medium (Thermo Fisher Australia Pty Ltd., Scoresby, Victoria, Australia) and 0.25 μg/ml Antibiotic Antimycotic Solution (Sigma Chemical Company, St. Louis, USA). The cells were allowed to propagate at 28 °C and 5% CO_2_. At approximately 80% confluency, cells were split into three T25 flasks for a further 3 to 4 passages before they were harvested by adding colcemid (0.05 μg/mL) for 3.5 h and treated with hypotonic solution (KCl, 0.075 mM). Slides were fixed with an ice-cold (ca 4 °C) 3:1 mixture of methanol and acetic acid. The cell suspension was dropped on to slides, air dried and frozen at − 80 °C until use. For DAPI (4^′^,6-diamidino-2-phenylindole) staining, each slide was mounted with anti-fade medium Vectashield (Vector Laboratories Inc., Burlingame, CA, USA) containing 1.5 mg/ml DAPI.

### Contig sequence analysis

To discover homologies of the male-specific contigs and identify any partial gene sequences that may exist, we used BLASTN to search each contig against representative reptilian and avian genomes available in Ensembl, Release 99 (*Anolis carolinensis*, *Crocodylus porosus*, *Gallus gallus*, *Pelodiscus sinensis*, *Podarcis muralis*, *Pogona vitticeps*, *Pseudonaja textilis*, *Notechis scutatus*, *Varanus komodoensis*, *Sphenodon punctatus*) with a minimum E-value of 0.000001 for reported alignments and a filter for low complexity regions. We used the same cut-off and filter to search the non-redundant database at the NCBI (https://blast.ncbi.nlm.nih.gov). The Dfam database [61] was used to search for known transposable elements.

## Supplementary information

**Additional file 1: Figure S1.** K-mer spectrum for the genome sequence of a male *B. duperreyi*. **Figure S2.** K-mer spectrum for the genome sequence of a female *B. duperreyi*. **Figure S3.** Number of Y enriched contigs ranging from 80 bp to 1374 bp resulting from the inchworm assembler. **Figure S4.** Contig length (bp) for the 92 subtraction contigs selected for PCR-based screening. **Figure S5.** Sequencing coverage for the 92 subtraction contigs selected for PCR-based screening. **Figure S6.** External and histological views of a) ovary b) testis in adult individuals of *B. duperreyi*. **Figure S7.** Karyotype of a male scincid lizard *Bassiana duperreyi*. **Figure S8.** Sequence alignment (a) and phylogeny (b) of bdM27_23_X5_798 contigs (top blue color highlight) with amplified 4 males (Piccadilly Circus_ACT) and 4 males (Anglesea _VIC). **Figure S9.** Sequence alignment (a) and phylogeny (b) of bdM27_10_X7_874 contigs (top blue color highlight) with amplified 4 males (Piccadilly Circus_ACT) and 4 males (Anglesea _VIC). **Figure S10.** Sequence alignment of bdM27_74_X11_649 contigs (top blue color highlight) with amplified 4 males (Piccadilly Circus_ACT) and 4 males (Anglesea _VIC). **Figure S11.** Sequence alignment (a) and phylogeny (b) of bdM27_82_X5_636 contigs (top blue color highlight) with amplified 2 males (Piccadilly Circus_ACT) and 4 males (Anglesea _VIC). **Figure S12.** Sequence alignment (a) and phylogeny (b) of bdM27_79_X5_643 contigs (top blue color highlight) with amplified 4 males (Piccadilly Circus_ACT) and 4 males (Anglesea _VIC). **Figure S13.** Sequence alignment (a) and phylogeny of bdM27_69_X9_658 contigs (top blue color highlight) with amplified 4 males (Piccadilly Circus_ACT) and 4 males (Anglesea _VIC). **Figure S14**. Sequence alignment of bdM27_87_X6_628 contigs (top blue color highlight) with amplified 4 males (Piccadilly Circus_ACT) and 4 males (Anglesea _VIC). **Table S1.** Estimates of evolutionary divergence between Piccadilly Circus and Anglesea individuals of *Bassiana duperryi*. **Table S2.** BLAST results for Y-specific contigs queried against representative reptile genomes. **Table S3.** Hits to known repeats in the Dfam database. **Table S4.** Specimen data, sex, locality and measurements for the *Bassiana duperreyi* specimens used in this study.

**Additional file 2: **Original gel images to accompany Figure 1 of the manuscript. **Figure S15.** Raw gel images for primer sets A. bdM27_10_X7_874, B. bdM27_87_X6_628, C. bdM27_23_X5_798, D. bdM27_69_X9_658, E. bdM27_74_X11_649, F. bdM27_79_X5_643, G. bdM27_82_X5_636. Each row represents alternating Male (band) and Female (no band) individuals (*n* = 20) spanned by ladders. Individuals from left to right in each gel are; 1st row – DDBD_8, DDBD_23, DDBD_9, DDBD_24, DDBD_12, DDBD_25, DDBD_13, DDBD_27, DDBD_14, DDBD_30, DDBD_16, DDBD_35, DDBD_18, DDBD_36, DDBD_19, DDBD_39, DDBD_21, DDBD_40, DDBD_22, DDBD_41; 2nd row DDBD_26, DDBD_47, DDBD_28, DDBD_56, DDBD_29, DDBD_57, DDBD_31, DDBD_59, DDBD_32, DDBD_60, DDBD_33, DDBD_62, DDBD_42, DDBD_100, DDBD_43, DDBD_287, DDBD_44, DDBD_288, DDBD_45, DDBD_289. Detailed specimen list available in the Additional file 1: Table S4.

## Data Availability

Data and materials are presented in the main paper and additional files, and in public repositories. The Sanger sequences of amplicons are available from Genbank (Accession Numbers MT756247–97). Illumina reads used as the basis of the k-mer analysis and subtraction are available from NCBI SRA repository (Accession Numbers SAMN15505254–5). The contigs obtained from the subtraction are available on the Dryad repository (10.5061/dryad.pvmcvdnj1), as are the perl scripts to undertake the subtraction and inchworm assembly.

## Abbreviations

GSD: Genotypic sex determination
TSD: Temperature dependent sex determination
AFLP: Amplified fragment length polymorphism
PCR: Polymerase chain reaction
DNA: Deoxyribonucleic acid
RAD-seq: Restriction Site-Associated DNA sequencing
ddRAD-seq: Double digest restriction-site associated DNA sequencing
DArT-seq: Diversity Arrays Technology sequencing
gRDA: Representational difference analysis
NCBI: The National Centre for Biotechnology Information
BLASTN: Basic local alignment search tool nucleotide

## References

[1] Düsing K (1884). Die Regulierung des geschlechtsverhä ltnisses bei der vermehrung der menschen, tiere und pflanzen. Jena Zschr Naturwise.

[2] Fisher RA. The genetical theory of natural selection. Oxford: Oxford University Press; 1930.

[3] Edwards AWF (2000). Carl Düsing (1884) on the regulation of the sex-ratio. Theor Popul Biol.

[4] Ohno S (1967). Sex chromosomes and sex-linked genes.

[5] Charlesworth D, Charlesworth B, Marais G (2005). Steps in the evolution of heteromorphic sex chromosomes. Heredity.

[6] Abbott JK, Nordén AK, Hansson B (2017). Sex chromosome evolution: historical insights and future perspectives. Proc R Soc B Biol Sci.

[7] Skaletsky H, Kuroda-Kawaguchi T, Minx PJ, Cordum HS, Hillier L, Brown LG, Repping S, Pyntikova T, Ali J, Bieri T, Chinwalla A (2003). The male-specific region of the human Y chromosome is a mosaic of discrete sequence classes. Nature.

[8] Ross MT, Grafham DV, Coffey AJ, Scherer S, McLay K, Muzny D, Platzer M, Howell GR, Burrows C, Bird CP, Frankish A (2005). The DNA sequence of the human X chromosome. Nature.

[9] Olmo E (2008). Trends in the evolution of reptilian chromosomes. Integr Comp Biol.

[10] Ezaz T, Moritz B, Waters P, Graves JA, Georges A, Sarre SD (2009). The ZW sex microchromosomes of an Australian dragon lizard share no homology with those of other reptiles or birds. Chromosom Res.

[11] O’Meally D, Ezaz T, Georges A, Sarre SD, Graves JA (2012). Are some chromosomes particularly good at sex? Insights from amniotes. Chromosom Res.

[12] Alam SM, Sarre SD, Gleeson D, Georges A, Ezaz T (2018). Did lizards follow unique pathways in sex chromosome evolution?. Genes.

[13] Deakin JE, Ezaz T (2019). Understanding the evolution of reptile chromosomes through applications of combined cytogenetics and genomics approaches. Cytogenet Genome Res.

[14] Ezaz T, Srikulnath K, Graves JA (2017). Origin of amniote sex chromosomes: an ancestral super-sex chromosome, or common requirements?. J Hered.

[15] Ezaz T, Quinn AE, Sarre SD, O’Meally D, Georges A, Graves JA (2009). Molecular marker suggests rapid changes of sex-determining mechanisms in Australian dragon lizards. Chromosom Res.

[16] PokornÁ M, Kratochvíl L (2009). Phylogeny of sex-determining mechanisms in squamate reptiles: are sex chromosomes an evolutionary trap?. Zool J Linnean Soc.

[17] Traut W, Eickhoff U, Schorch JC, Sharma AK, Sharma A (2001). Identification and analysis of sex chromosomes by comparative genomic hybridization (CGH). Chromosome painting.

[18] Ezaz T, Quinn AE, Miura I, Sarre SD, Georges A, Graves JA (2005). The dragon lizard *Pogona vitticeps* has ZZ/ZW micro-sex chromosomes. Chromosom Res.

[19] Wright AE, Dean R, Zimmer F, Mank JE (2016). How to make a sex chromosome. Nat Commun.

[20] Guillon H, de Massy B (2002). An initiation site for meiotic crossing-over and gene conversion in the mouse. Nat Genet.

[21] Jeffreys AJ, Kauppi L, Neumann R (2001). Intensely punctate meiotic recombination in the class II region of the major histocompatibility complex. Nat Genet.

[22] Kikuchi K, Kai W, Hosokawa A, Mizuno N, Suetake H, Asahina K, Suzuki Y (2007). The sex-determining locus in the tiger pufferfish, *Takifugu rubripes*. Genetics.

[23] Kamiya T, Kai W, Tasumi S, Oka A, Matsunaga T, Mizuno N, Fujita M, Suetake H, Suzuki S, Hosoya S, Tohari S (2012). A trans-species missense SNP in Amhr2 is associated with sex determination in the tiger pufferfish, *Takifugu rubripes* (fugu). PLoS Genet.

[24] Palaiokostas C, Bekaert M, Khan MG, Taggart JB, Gharbi K, McAndrew BJ, Penman DJ (2013). Mapping and validation of the major sex-determining region in Nile tilapia (*Oreochromis niloticus L*.) using RAD sequencing. PLoS One.

[25] Gamble T, Zarkower D (2014). Identification of sex-specific molecular markers using restriction site-associated DNA sequencing. Mol Ecol Resour.

[26] Shi X, Waiho K, Li X, Ikhwanuddin M, Miao G, Lin F, Zhang Y, Li S, Zheng H, Liu W, Aweya JJ (2018). Female-specific SNP markers provide insights into a WZ/ZZ sex determination system for mud crabs *Scylla paramamosain*, *S. tranquebarica* and *S. serrata* with a rapid method for genetic sex identification. BMC Genomics.

[27] Welsh J, McClelland M (1990). Fingerprinting genomes using PCR with arbitrary primers. Nucleic Acids Res.

[28] Williams JGKK, Kubelik AR, Livak KJ, Rafalski JA, Tingey SV (1990). DNA polymorphisms amplified by arbitrary primers are useful as genetic markers. Nucleic Acids Res.

[29] Martinez EA, Destombe C, Quillet MC, Valero M (1999). Identification of random amplified polymorphic DNA (RAPD) markers highly linked to sex determination in the red alga *Gracilaria gracilis*. Mol Ecol.

[30] Griffiths RI, Orr KA (1999). The use of amplified fragment length polymorphism (AFLP) in the isolation of sex-specific markers. Mol Ecol.

[31] Griffiths R, Baker AJ (2000). Sex identification using DNA markers. Molecular methods in ecology.

[32] Quinn AE, Radder RS, Sarre SD, Georges A, Ezaz T, Shine R (2009). Isolation and development of a molecular sex marker for *Bassiana duperreyi*, a lizard with XX/XY sex chromosomes and temperature-induced sex reversal. Mol Genet Genomics.

[33] Ayers KL, Davidson NM, Demiyah D, Roeszler KN, Grützner F, Sinclair AH, Oshlack A, Smith CA (2013). RNA sequencing reveals sexually dimorphic gene expression before gonadal differentiation in chicken and allows comprehensive annotation of the W-chromosome. Genome Biol.

[34] Chen N, Bellott DW, Page DC, Clark AG (2012). Identification of avian W-linked contigs by short-read sequencing. BMC Genomics.

[35] Bidon T, Schreck N, Hailer F, Nilsson MA, Janke A (2015). Genome-wide search identifies 1.9 Mb from the polar bear Y chromosome for evolutionary analyses. Genome Biol Evol.

[36] Gamble T, Geneva AJ, Glor RE, Zarkower D (2014). Anolis sex chromosomes are derived from a single ancestral pair. Evolution.

[37] Peterson BK, Weber JN, Kay EH, Fisher HS, Hoekstra HE (2012). Double digest RADseq: an inexpensive method for de novo SNP discovery and genotyping in model and non-model species. PLoS One.

[38] Carmichael SN, Bekaert M, Taggart JB, Christie HR, Bassett DI, Bron JE, Skuce PJ, Gharbi K, Skern-Mauritzen R, Sturm A (2013). Identification of a sex-linked SNP marker in the salmon louse (*Lepeophtheirus salmonis*) using RAD sequencing. PLoS One.

[39] Brown JK, Taggart JB, Bekaert M, Wehner S, Palaiokostas C, Setiawan AN, Symonds JE, Penman DJ (2016). Mapping the sex determination locus in the hāpuku (*Polyprion oxygeneios*) using ddRAD sequencing. BMC Genomics.

[40] Fowler BLS, Buonaccorsi VP (2016). Genomic characterization of sex-identification markers in *Sebastes carnatus* and *Sebastes chrysomelas* rockfishes. Mol Ecol.

[41] Gamble T, Coryell J, Ezaz T, Lynch J, Scantlebury DP, Zarkower D (2015). Restriction site-associated DNA sequencing (RAD-seq) reveals an extraordinary number of transitions among gecko sex-determining systems. Mol Biol Evol.

[42] Hime PM, Briggler JT, Reece JS, Weisrock DW (2019). Genomic data reveal conserved female heterogamety in giant salamanders with gigantic nuclear genomes. G3.

[43] Luo W, Xia Y, Yue B, Zeng X (2020). Assigning the sex-specific markers via genotyping-by-sequencing onto the Y chromosome for a torrent frog *Amolops mantzorum*. Genes.

[44] Kilian A, Wenzl P, Huttner E, Carling J, Xia L, Blois H, Caig V, Heller-Uszynska K, Jaccoud D, Hopper C, Aschenbrenner-Kilian M (2012). Diversity arrays technology: a generic genome profiling technology on open platforms. Methods Mol Biol.

[45] Lambert MR, Skelly DK, Ezaz T (2016). Sex-linked markers in the north American green frog (*Rana clamitans*) developed using DArTseq provide early insight into sex chromosome evolution. BMC Genomics.

[46] Sopniewski J, Shams F, Scheele BC, Kefford BJ, Ezaz T (2019). Identifying sex-linked markers in *Litoria aurea*: a novel approach to understanding sex chromosome evolution in an amphibian. Sci Rep.

[47] Lowry DB, Hoban S, Kelley JL, Lotterhos KE, Reed LK, Antolin MF, Storfer A (2017). Breaking RAD: an evaluation of the utility of restriction site-associated DNA sequencing for genome scans of adaptation. Mol Ecol Resour.

[48] Hollestelle A, Schutte M (2005). Representational difference analysis as a tool in the search for new tumor suppressor genes. Methods Mol Med.

[49] Sakharkar KR, Sakharkar MK, Chow VT (2004). A novel genomics approach for the identification of drug targets in pathogens, with special reference to *Pseudomonas aeruginosa*. In Silico Biol.

[50] Dutta A, Singh SK, Ghosh P, Mukherjee R, Mitter S, Bandyopadhyay D (2006). *In silico* identification of potential therapeutic targets in the human pathogen helicobacter pylori. In Silico Biol.

[51] Chong CE, Lim BS, Nathan S, Mohamed R (2006). *In silico* analysis of *Burkholderia pseudomallei* genome sequence for potential drug targets. In Silico Biol.

[52] Isakov O, Modai S, Shomron N (2011). Pathogen detection using short-RNA deep sequencing subtraction and assembly. Bioinformatics.

[53] Kawakami Y, Fujita T, Matsuzaki Y, Sakurai T, Tsukamoto M, Toda M, Sumimoto H (2004). Identification of human tumor antigens and its implications for diagnosis and treatment of cancer. Cancer Sci.

[54] Nishimura Y, Tomita Y, Yuno A, Yoshitake Y, Shinohara M (2015). Cancer immunotherapy using novel tumor-associated antigenic peptides identified by genome-wide cDNA microarray analyses. Cancer Sci.

[55] Kakimi K, Karasaki T, Matsushita H, Sugie T (2017). Advances in personalized cancer immunotherapy. Breast Cancer.

[56] Mirvish ED, Shuda M (2016). Strategies for human tumor virus discoveries: from microscopic observation to digital transcriptome subtraction. Front Microbiol.

[57] Donnellan SC (1985). The evolution of sex chromosomes in scincid lizards.

[58] Shine R, Elphick MJ, Donnellan S (2002). Co-occurrence of multiple, supposedly incompatible modes of sex determination in a lizard population. Ecol Lett.

[59] Radder RS, Quinn AE, Georges A, Sarre SD, Shine R (2008). Genetic evidence for co-occurrence of chromosomal and thermal sex determining systems in a lizard. Biol Lett.

[60] Nguyen NT, Vincens P, Roest Crollius H, Louis A (2018). Genomicus 2018: karyotype evolutionary trees and on-the-fly synteny computing. Nucleic Acids Res.

[61] Wheeler TJ, Clements J, Eddy SR, Hubley R, Jones TA, Jurka J, Smit AF, Finn RD (2012). Dfam: a database of repetitive DNA based on profile hidden Markov models. Nucleic Acids Res.

[62] Dubey S, Shine R (2010). Evolutionary diversification of the lizard genus *Bassiana* (Scincidae) across southern Australia. PLoS One.

[63] Taberlet P, Waits LP, Luikart G (1999). Noninvasive genetic sampling: look before you leap. Trends Ecol Evol.

[64] Ferguson-Smith M (2007). The evolution of sex chromosomes and sex determination in vertebrates and the key role of DMRT1. Sex Dev.

[65] Rovatsos M, Kratochvíl L (2017). Molecular sexing applicable in 4000 species of lizards and snakes? From dream to real possibility. Methods Ecol Evol.

[66] Boulanger J, White GC, Proctor M, Stenhouse G, Machutchon G, Himmer S (2008). Use of occupancy models to estimate the influence of previous live captures on DNA-based detection probabilities of grizzly bears. J Wildl Manag.

[67] Dawson DA, Bird S, Horsburgh GJ, Ball AD (2015). Autosomal and Z-linked microsatellite markers enhanced for cross-species utility and assessed in a range of birds, including species of conservation concern. Conserv Genet Resour.

[68] Literman R, Radhakrishnan S, Tamplin J, Burke R, Dresser C, Valenzuela N (2017). Development of sexing primers in *Glyptemys insculpta* and *Apalone spinifera* turtles uncovers an XX/XY sex-determining system in the critically-endangered bog turtle *Glyptemys muhlenbergii*. Conserv Genet Resour.

[69] Zhang L, Ma X, Jiang J, Lu X (2012). Stronger condition dependence in female size explains altitudinal variation in sexual size dimorphism of a Tibetan frog. Biol J Linn Soc.

[70] Sulandart S, Zein MSA (2012). Application of two molecular sexing methods for Indonesian bird species: implication for captive breeding programs in Indonesia. HAYATI J Biosci.

[71] Fang S, Zhang Y, Shi X, Zheng H, Li S, Zhang Y, Fazhan H, Waiho K, Tan H, Ikhwanuddin M, Ma H (2020). Identification of male-specific SNP markers and development of PCR-based genetic sex identification technique in crucifix crab (*Charybdis feriatus*) with implication of an XX/XY sex determination system. Genomics.

[72] Zheng S, Wang X, Zhang S, Long J, Tao W, Li M, Wang D (2020). Screening and characterization of sex-linked DNA markers and marker-assisted selection in the southern catfish (*Silurus meridionalis*). Aquaculture.

[73] Quinn AE, Sarre SD, Ezaz T, Marshall Graves JA, Georges A (2011). Evolutionary transitions between mechanisms of sex determination in vertebrates. Biol Lett.

[74] Dash HR, Rawat N, Das S (2020). Alternatives to amelogenin markers for sex determination in humans and their forensic relevance. Mol Biol Rep.

[75] Holleley CE, Sarre SD, O'Meally D, Georges A (2016). Sex reversal in reptiles: reproductive oddity or powerful driver of evolutionary change?. Sex Dev.

[76] Holleley CE, O'Meally D, Sarre SD, Graves JA, Ezaz T, Matsubara K, Azad B, Zhang X, Georges A (2015). Sex reversal triggers the rapid transition from genetic to temperature-dependent sex. Nature.

[77] Whiteley SL, Holleley CE, Ruscoe WA, Castelli M, Whitehead DL, Lei J, Georges A, Weisbecker V (2017). Sex determination mode does not affect body or genital development of the central bearded dragon (*Pogona vitticeps*). Evo Devo.

[78] Whiteley SL, Weisbecker V, Georges A, Gauthier AR, Whitehead DL, Holleley CE (2018). Developmental asynchrony and antagonism of sex determination pathways in a lizard with temperature-induced sex reversal. Sci Rep.

[79] Cortez D, Marin R, Toledo-Flores D, Froidevaux L, Liechti A, Waters PD, Gruetzner F, Kaessmann H (2014). Origins and functional evolution of Y chromosomes across mammals. Nature.

[80] Cornejo-Páramo P, Dissanayake DSB, Lira-Noriega A, Martínez-Pacheco ML, Acosta A, Ramírez-Suástegui C, Méndez-de-la-Cruz FR, Székely T, Urrutia AO, Georges A, Cortez D (2020). Viviparous reptile regarded to have temperature-dependent sex determination has old XY chromosomes. Genome Biol Evol.

[81] Seufert W, Jentsch S (1990). Ubiquitin-conjugating enzymes UBC4 and UBC5 mediate selective degradation of short-lived and abnormal proteins. EMBO J.

[82] Wing SS, Bedard N, Morales C, Hingamp P, Trasler J (1996). A novel rat homolog of the Saccharomyces cerevisiae ubiquitin-conjugating enzymes UBC4 and UBC5 with distinct biochemical features is induced during spermatogenesis. Mol Cell Biol.

[83] Yokota N, Harada Y, Sawada H (2010). Identification of testis-specific ubiquitin-conjugating enzyme in the ascidian *Ciona intestinalis*. Mol Reprod Dev.

[84] Schwanz LE, Georges A, Holleley CE, Sarre S (2020). Climate change, sex reversal and lability of sex determining systems. J Evol Biol.

[85] Cogger HG. Reptiles and amphibians of Australia. 7th ed. Canberra: CSIRO Publishing; 2014.

[86] Harlow PS. A harmless technique for sexing hatchiling lizards. Herpetol Rev. 1996;27:71–2.

[87] Ezaz T, O’Meally D, Quinn AE, Sarre SD, Georges A, Graves JA (2008). A simple non-invasive protocol to establish primary cell lines from tail and toe explants for cytogenetic studies in Australian dragon lizards (Squamata: Agamidae). Cytotechnology.

[88] Marçais G, Kingsford C (2011). A fast, lock-free approach for efficient parallel counting of occurrences of k-mers. Bioinformatics.

[89] Untergasser A, Cutcutache I, Koressaar T, Ye J, Faircloth BC, Remm M, Rozen SG (2012). Primer3—new capabilities and interfaces. Nucleic Acids Res.

[90] Kearse M, Moir R, Wilson A, Stones-Havas S, Cheung M, Sturrock S, Buxton S, Cooper A, Markowitz S, Duran C, Thierer T (2012). Geneious basic: an integrated and extendable desktop software platform for the organization and analysis of sequence data. Bioinformatics.

[91] Doddamani LS (1994). Histoenzymological studies on embryonic and posthatching development of the ovary in the tropical oviparous lizard, *Calotes versicolor*. J Morphol.

[92] Doddamani LS (2006). Differentiation and development of testis in the oviparous lizard, *Calotes versicolor* (Daud.). J Exp Zoolog Part A Comp Exp Biol.

