# Supplementary material for: Identification of Y chromosome markers in the eastern three-lined skink (Bassiana duperreyi) using in silico whole genome subtraction
Source: BMC Genomics. 2020 Sep 29;21:667. doi: 10.1186/s12864-020-07071-2 (PMC7526180; doi:10.1186/s12864-020-07071-2)
Supplement: Supplementary file 1 — Additional file 1: Figure S1. K-mer spectrum for the genome sequence of a male B. duperreyi. Figure S2. K-mer spectrum for the genome sequence of a female B. duperreyi. Figure S3. Number of Y enriched contigs ranging from 80 bp to 1374 bp resulting from the inchworm assembler. Figure S4. Contig length (bp) for the 92 subtraction contigs selected for PCR-based screening. Figure S5. Sequencing coverage for the 92 subtraction contigs selected for PCR-based screening. Figure S6. External and histological views of a) ovary b) testis in adult individuals of B. duperreyi. Figure S7. Karyotype of a male scincid lizard Bassiana duperreyi. Figure S8. Sequence alignment (a) and phylogeny (b) of bdM27_23_X5_798 contigs (top blue color highlight) with amplified 4 males (Piccadilly Circus_ACT) and 4 males (Anglesea _VIC). Figure S9. Sequence alignment (a) and phylogeny (b) of bdM27_10_X7_874 contigs (top blue color highlight) with amplified 4 males (Piccadilly Circus_ACT) and 4 males (Anglesea _VIC). Figure S10. Sequence alignment of bdM27_74_X11_649 contigs (top blue color highlight) with amplified 4 males (Piccadilly Circus_ACT) and 4 males (Anglesea _VIC). Figure S11. Sequence alignment (a) and phylogeny (b) of bdM27_82_X5_636 contigs (top blue color highlight) with amplified 2 males (Piccadilly Circus_ACT) and 4 males (Anglesea _VIC). Figure S12. Sequence alignment (a) and phylogeny (b) of bdM27_79_X5_643 contigs (top blue color highlight) with amplified 4 males (Piccadilly Circus_ACT) and 4 males (Anglesea _VIC). Figure S13. Sequence alignment (a) and phylogeny of bdM27_69_X9_658 contigs (top blue color highlight) with amplified 4 males (Piccadilly Circus_ACT) and 4 males (Anglesea _VIC). Figure S14. Sequence alignment of bdM27_87_X6_628 contigs (top blue color highlight) with amplified 4 males (Piccadilly Circus_ACT) and 4 males (Anglesea _VIC). Table S1. Estimates of evolutionary divergence between Piccadilly Circus and Anglesea individuals of Bassiana duperryi. Table S2. [file 12864_2020_7071_MOESM1_ESM.pdf]

Supplemental Materials

Figures S1-S14, Tables S1-S4

Dissanayake, D.S.B., Holleley, C.E., Hill, L., O'Meally, D., Deakin, J., and Georges, A. (2020). Identification of Y chromosome markers in the eastern three-lined skink (*Bassiana duperreyi*) using in silico whole genome subtraction. BMC Genomics, in press.

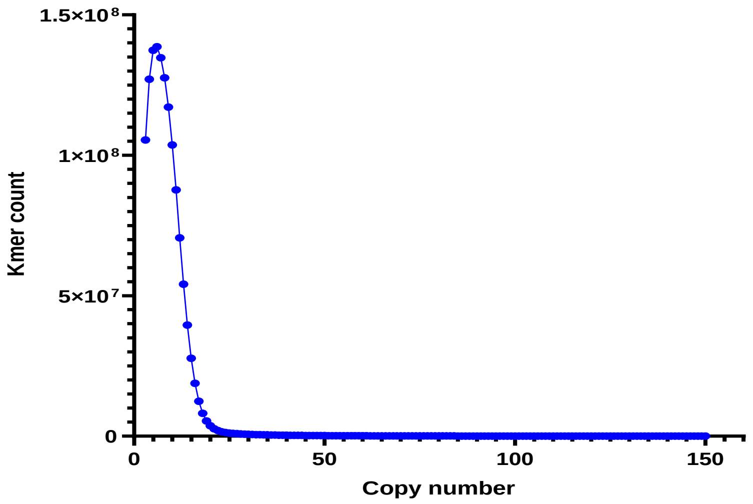

Figure S1. K-mer spectrum for the genome sequence of a male *Bassiana duperreyi*. The Illumina run yielded 96.7 Gb of clean data, which generated 14,310,783,435 K-mer sequences.

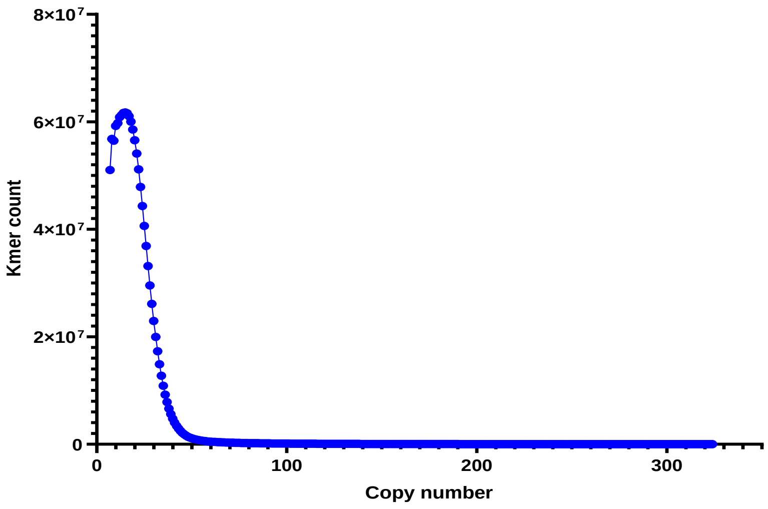

Figure S2. K-mer spectrum for the genome sequence of a female *Bassiana duperreyi*. The Illumina run yielded 81.41 Gb of clean data, which generated 36,695,139,446 k-mer sequences.

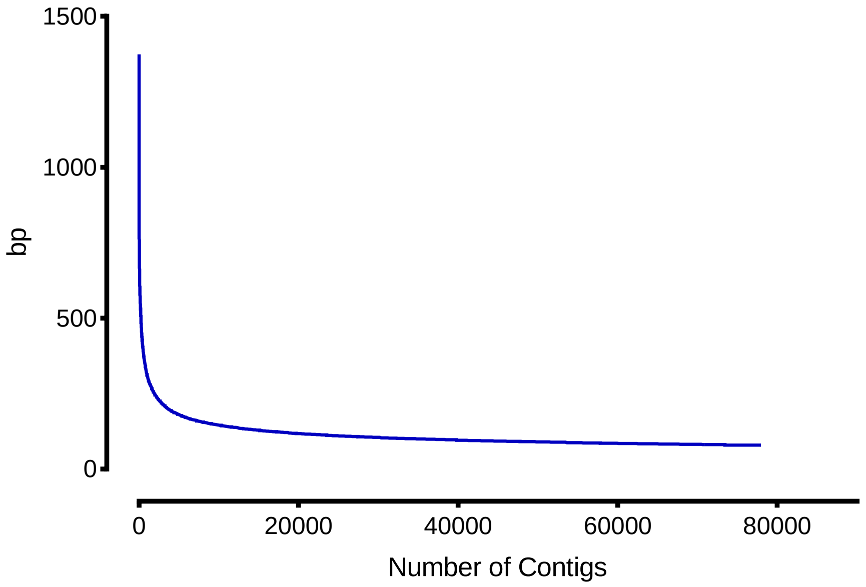

Figure S3. Number of Y enriched contigs ranging from 80 bp to 1374 bp resulting from the inchworm assembler.

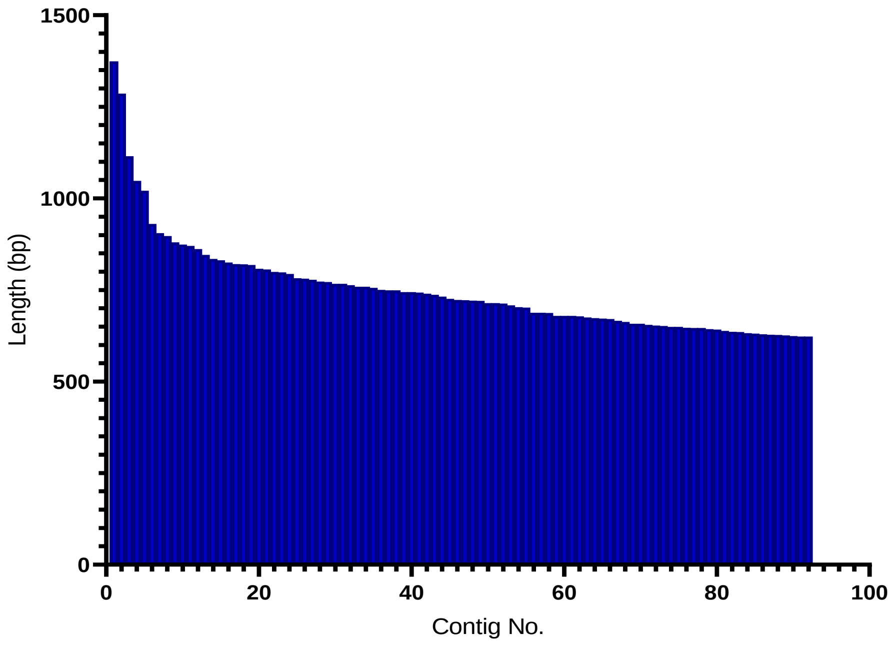

Figure S4. Contig length (bp) for the 92 subtraction contigs selected for PCR-based screening.

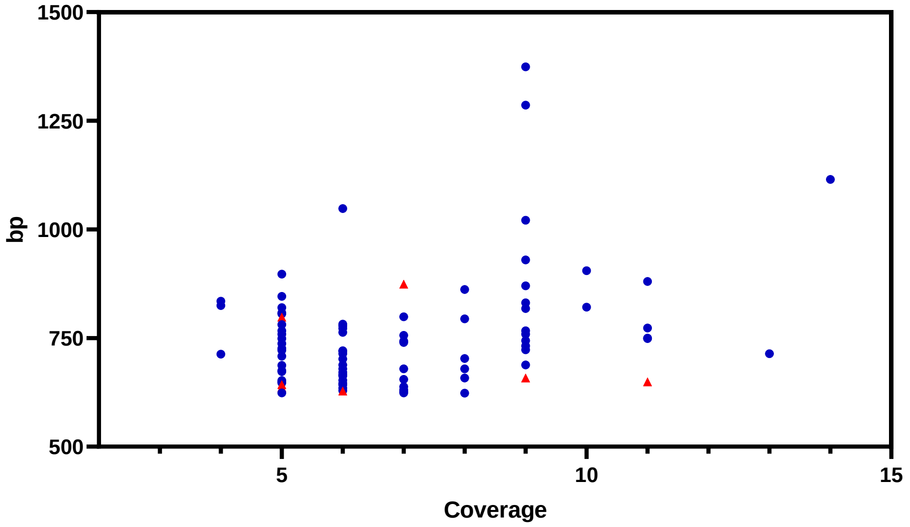

Figure S5. Sequencing coverage for the 92 subtraction contigs selected for PCR-based screening. Red triangles indicate the seven confirmed Y-chromosome subtraction contigs. Blue circles indicate contigs that did not pass PCR validation with a sex-specific pattern of amplification.

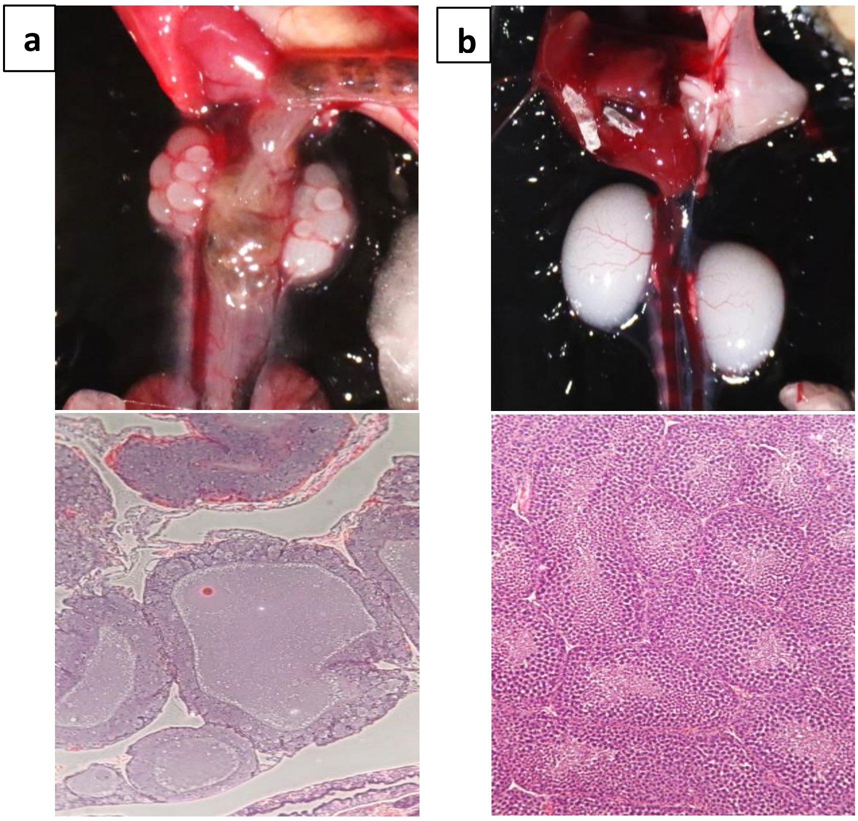

Figure S6. External and histological views of a) ovary b) testis in adult individuals of *B. duperreyi*.

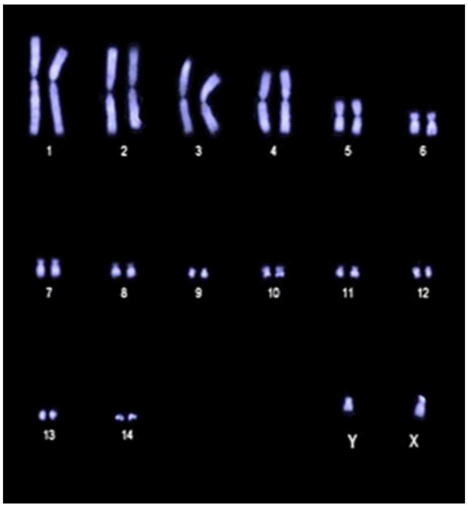

Figure S7. Karyotype of a male scincid lizard *Bassiana duperreyi*.

Figure S8. Sequence alignment (a) and phylogeny (b) of bdM27_23_X5_798 contigs (top blue color highlight) with amplified 4 males (Piccadilly Circus_ACT) and 4 males (Anglesea _VIC). Sequences were aligned with Geneious R10.2.6. The results show differences in nucleotide sites, suggesting that Y chromosome region is divergent in each population. Dash indicate gaps and sequences highlighted in colors denote mismatches. Tree is a phylogenetic analysis of sequence variation between ACT and VIC populations. The evolutionary history was inferred by using the Maximum Likelihood method based on the Kimura 2-parameter model [1]. Bootstrap support values the number of substitutions per site. All position with gaps and missing data have been eliminated. The analysis involved 8 nucleotide sequences. There were a total of 377 positions in the final dataset. Evolutionary analyses were conducted in MEGA7[2].

a)

b)

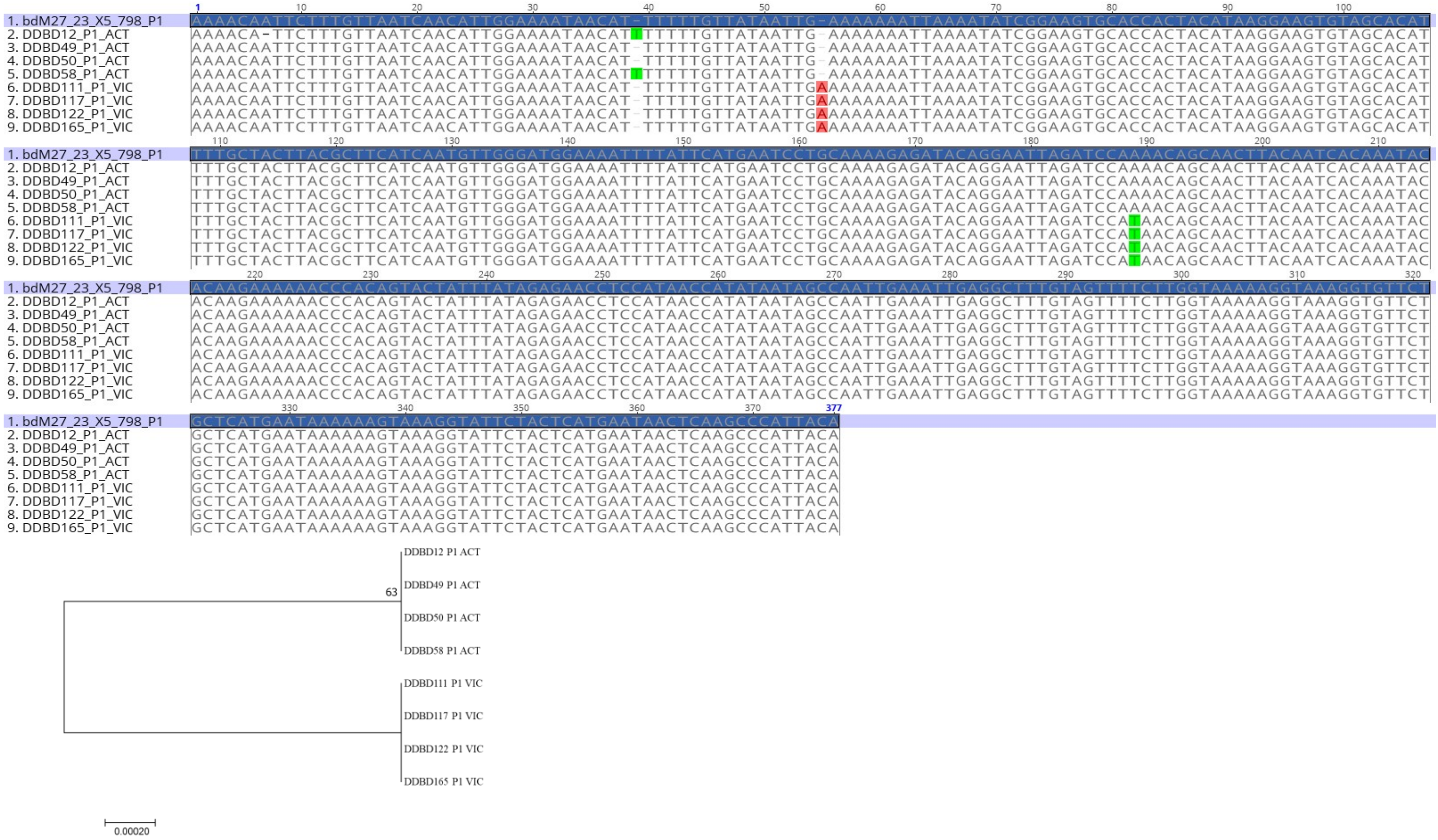

Figure S9. Sequence alignment (a) and phylogeny (b) of bdM27_10_X7_874 contigs (top blue color highlight) with amplified 2 males (Piccadilly Circus_ACT) and a male (Anglesea _VIC). Sequences were aligned with Geneious R10.2.6. The results show differences in nucleotide sites, suggesting that Y chromosome region is divergent in each population. Dash indicate gaps and sequences highlighted in colors denote mismatches. Tree is a phylogenetic analysis of sequence variation between ACT and VIC populations. The evolutionary history was inferred by using the Maximum Likelihood method based on the Hasegawa-Kishino-Yano mode [3]. Bootstrap support values the number of substitutions per site. All position with gaps and missing data have been eliminated. The analysis involved 5 nucleotide sequences. There was a total of 340 positions in the final dataset. Evolutionary analyses were conducted in MEGA7 [2].

a)

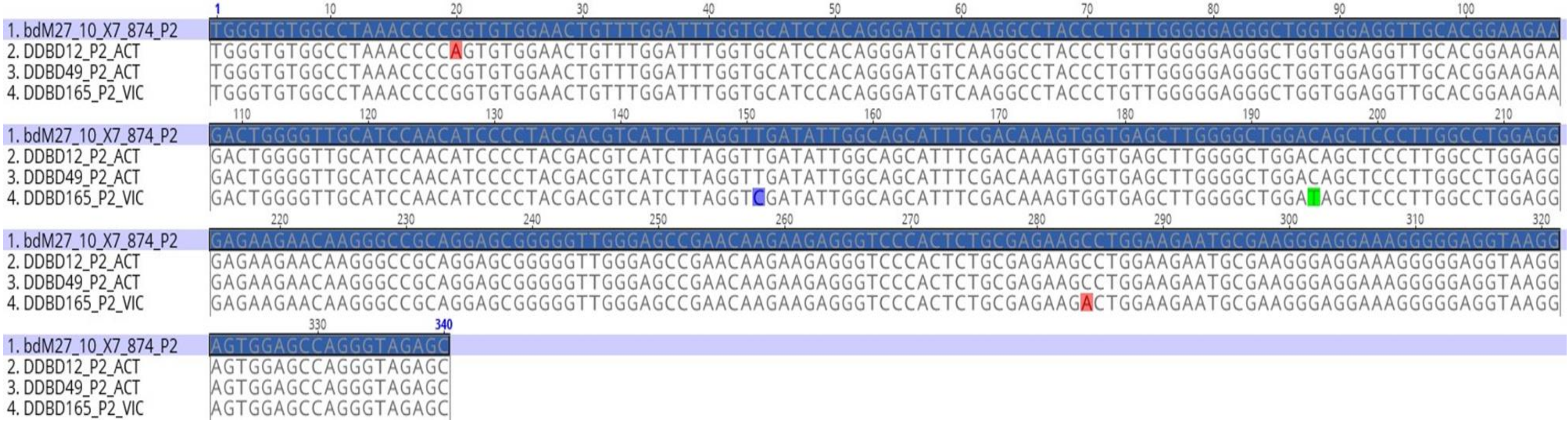

b)

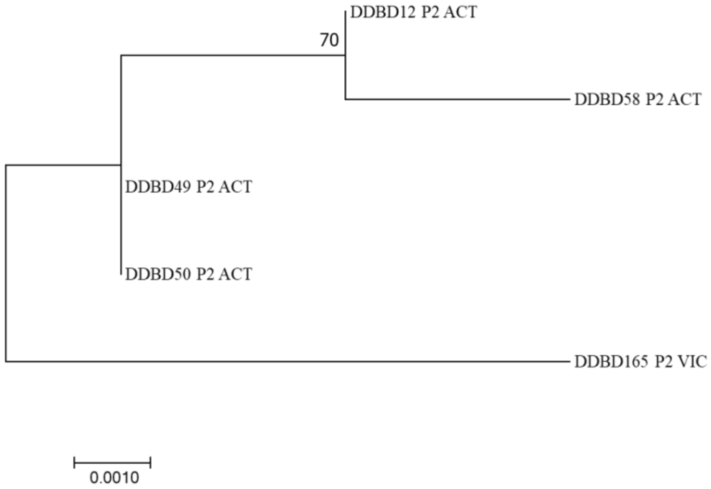

Figure S10. Sequence alignment of bdM27_74_X11_649 contigs (top blue color highlight) with amplified 3 males (Piccadilly Circus_ACT) and 4 males (Anglesea _VIC). Sequences were aligned with Geneious R10.2.6. No differences found in sequences.

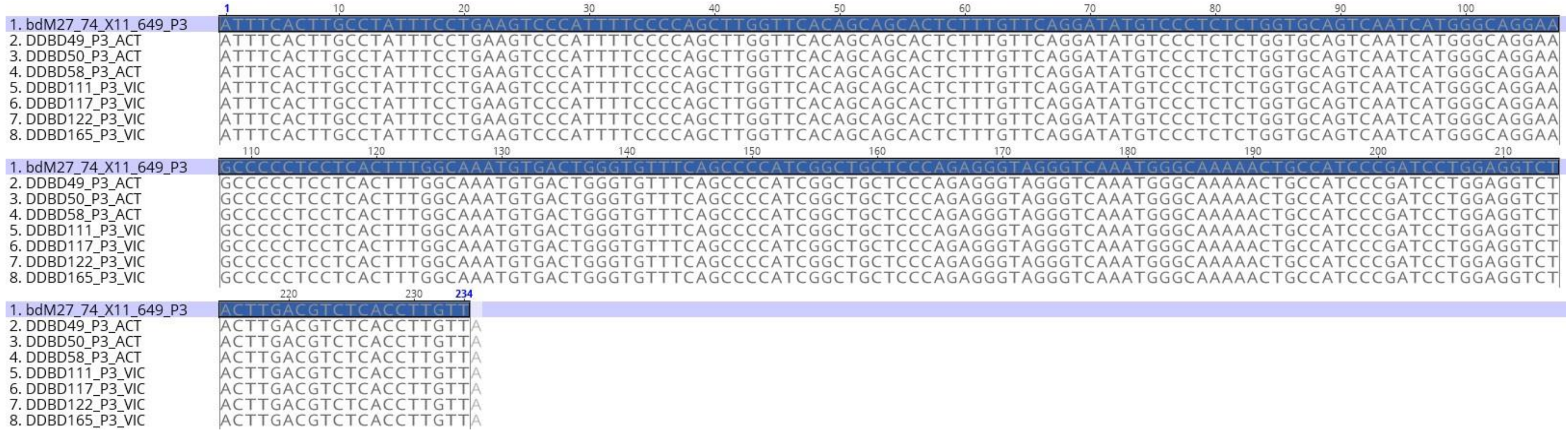

Figure S11. Sequence alignment (a) and phylogeny (b) of bdM27_82_X5_636 contigs (top blue color highlight) with amplified 2 males (Piccadilly Circus_ACT) and 4 males (Anglesea _VIC). Sequences were aligned with Geneious R10.2.6. The results show differences in nucleotide sites, suggesting that Y chromosome region is divergent in each population. Dash indicate gaps and sequences highlighted in colors denote mismatches. Tree is a phylogenetic analysis of sequence variation between ACT and VIC populations. The evolutionary history was inferred by using the Maximum Likelihood method based on the Jukes-Cantor model [4]. Bootstrap support values the number of substitutions per site. All position with gaps and missing data have been eliminated. The analysis involved 7 nucleotide sequences. There were a total of 251 positions in the final dataset. Evolutionary analyses were conducted in MEGA7 [2].

a)

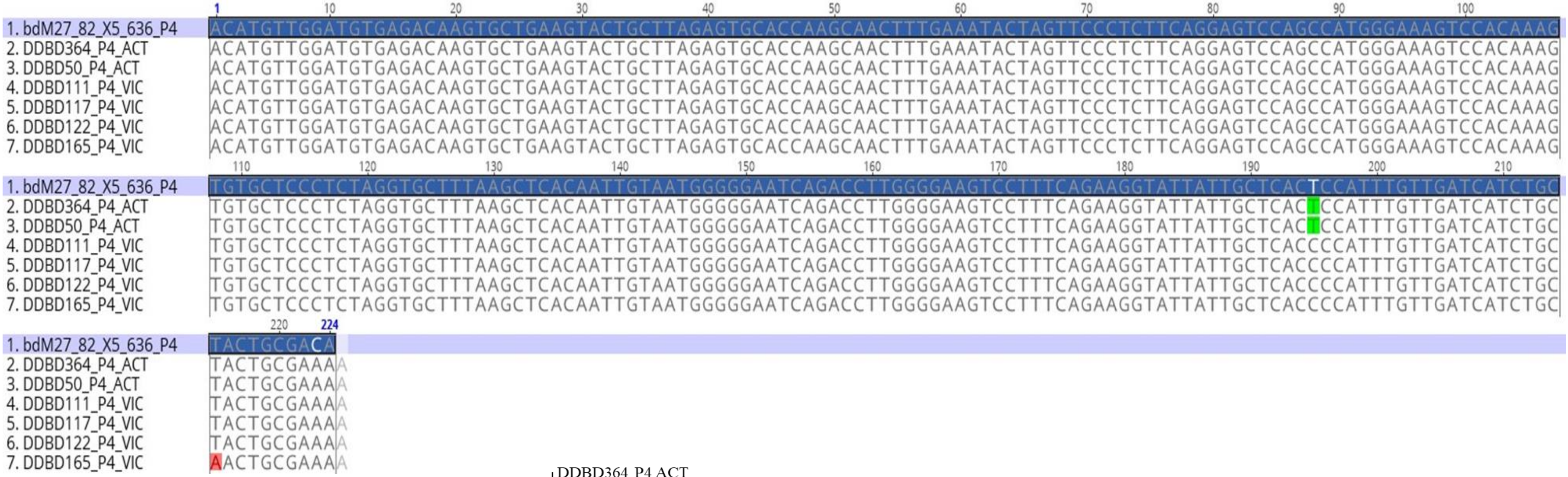

b)

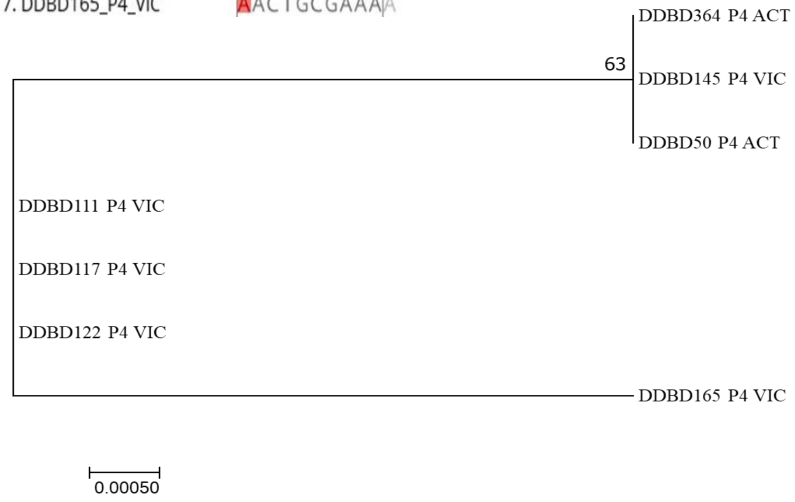

Figure S12. Sequence alignment (a) and phylogeny (b) of bdM27_79_X5_643 contigs (top blue color highlight) with amplified 4 males (Piccadilly Circus_ACT) and 4 males (Anglesea _VIC). Sequences were aligned with Geneious R10.2.6. The results show differences in nucleotide sites, suggesting that Y chromosome region is divergent in each population. Dash indicate gaps and sequences highlighted in colors denote mismatches. Tree is a phylogenetic analysis of sequence variation between ACT and VIC populations. The evolutionary history was inferred by using the Maximum Likelihood method based on the Jukes-Cantor model [4]. Bootstrap support values the number of substitutions per site. All position with gaps and missing data have been eliminated. The analysis involved 8 nucleotide sequences. There were a total of 266 positions in the final dataset. Evolutionary analyses were conducted in MEGA7 [2].

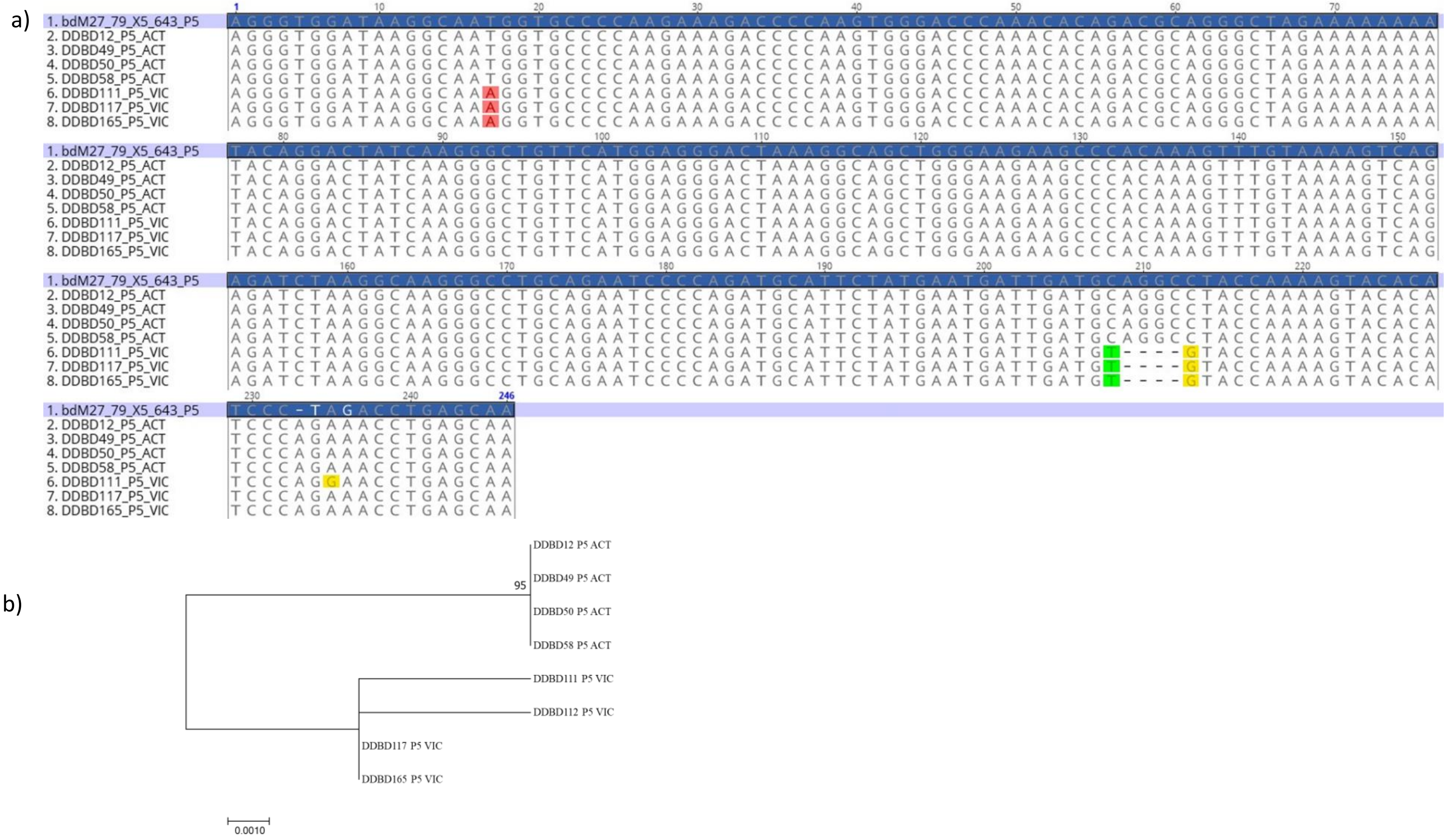

Figure S13. Sequence alignment (a) and phylogeny of bdM27_69_X9_658 contigs (top blue color highlight) with amplified 4 males (Piccadilly Circus_ACT) and 4 males (Anglesea _VIC). Sequences were aligned with Geneious R10.2.6. The results show differences in nucleotide sites, suggesting that Y chromosome region is divergent in each population. Dash indicate gaps and sequences highlighted in colors denote mismatches. Tree is a phylogenetic analysis of sequence variation between ACT and VIC populations using bdM27_69_X9_658. The evolutionary history was inferred by using the Maximum Likelihood method based on the Hasegawa-Kishino-Yano model [3]. Bootstrap support values the number of substitutions per site. All position with gaps and missing data have been eliminated. The analysis involved 8 nucleotide sequences. There were a total of 367 positions in the final dataset. Evolutionary analyses were conducted in MEGA7.

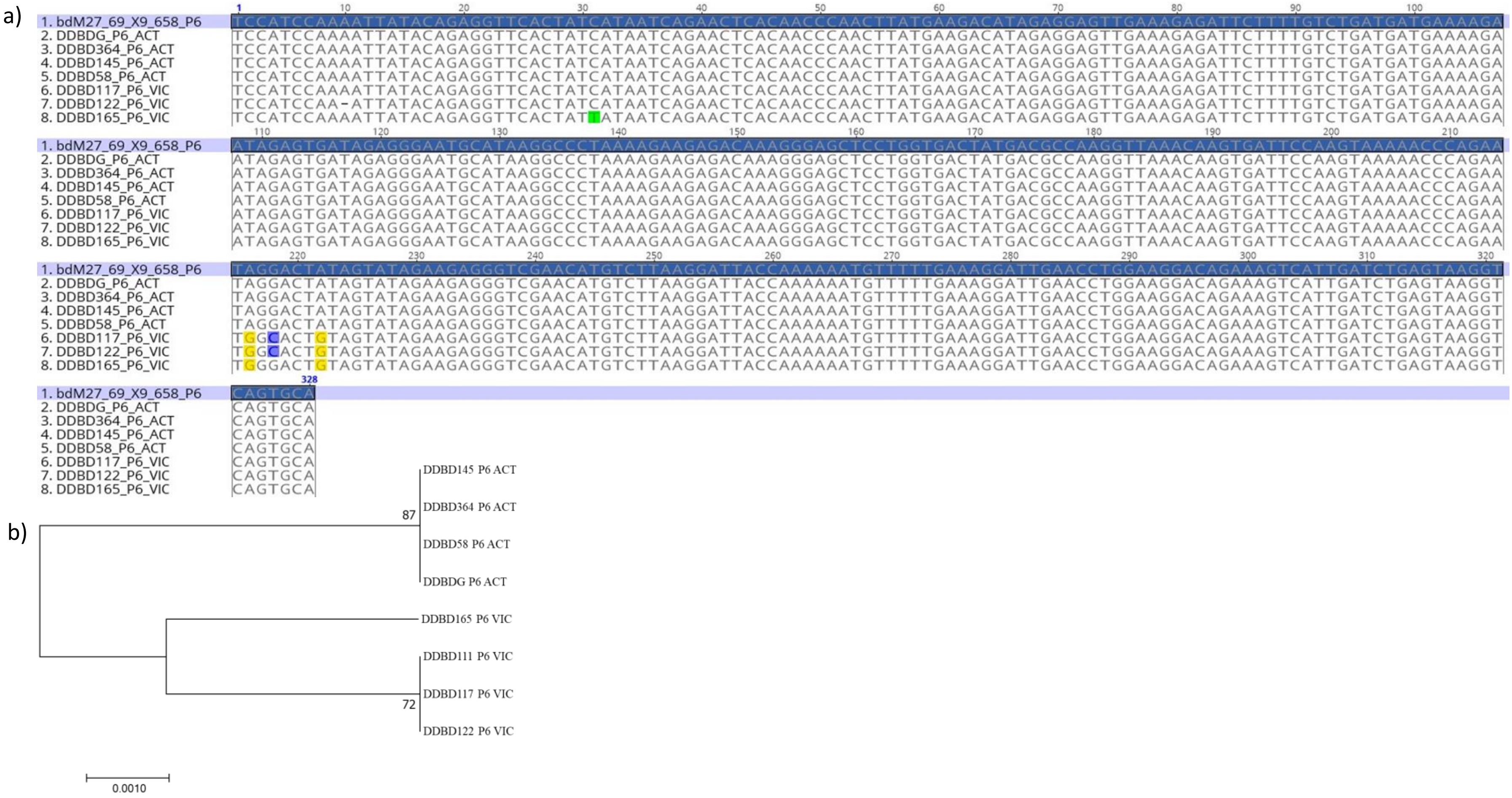

Figure S14. Sequence alignment of bdM27_87_X6_628 contigs (top blue color highlight) with amplified 4 males (Piccadilly Circus_ACT) and 4 males (Anglesea _VIC). Sequences were aligned with Geneious R10.2.6. No differences found in sequences.

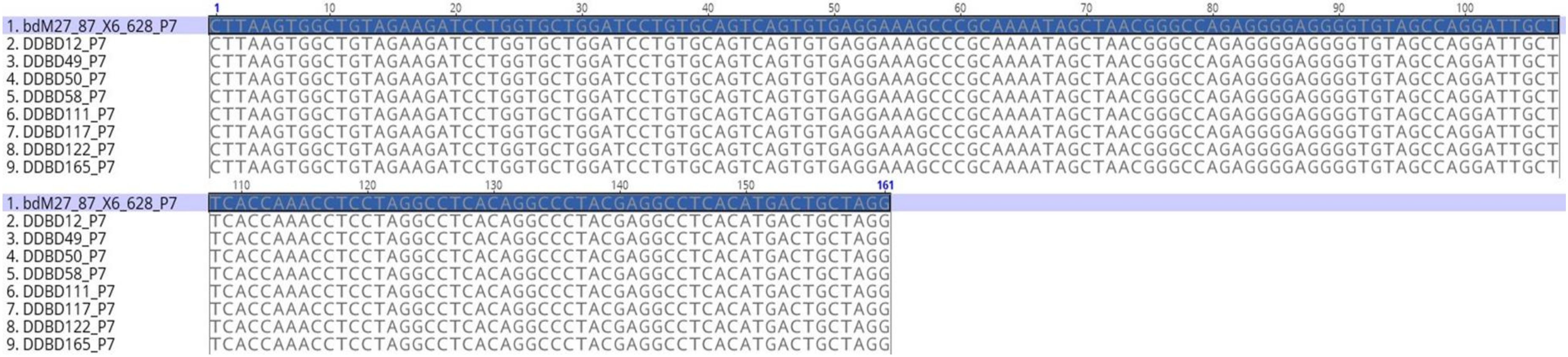

Table S1. Estimates of evolutionary divergence between Piccadilly Circus and Anglesea individuals of *Bassiana duperreyi*. The number of base substitutions per site from between sequences are shown. Analyses were conducted using the Maximum Composite Likelihood model [5]. All positions containing gaps and missing data were eliminated. Evolutionary analyses were conducted in MEGA7 [2].

Amplified contigs name	Piccadilly Circus Population	Anglesea Population	Genetic Distance
bdM27_23_X5_798	DDBD12 P1 ACT	DDBD111 P1 VIC	0.003
	DDBD49 P1 ACT	DDBD111 P1 VIC	0.003
	DDBD50 P1 ACT	DDBD111 P1 VIC	0.003
	DDBD58 P1 ACT	DDBD111 P1 VIC	0.003
	DDBD12 P1 ACT	DDBD117 P1 VIC	0.003
	DDBD49 P1 ACT	DDBD117 P1 VIC	0.003
	DDBD50 P1 ACT	DDBD117 P1 VIC	0.003
	DDBD58 P1 ACT	DDBD117 P1 VIC	0.003
	DDBD12 P1 ACT	DDBD122 P1 VIC	0.003
	DDBD49 P1 ACT	DDBD122 P1 VIC	0.003
	DDBD50 P1 ACT	DDBD122 P1 VIC	0.003
	DDBD58 P1 ACT	DDBD122 P1 VIC	0.003
	DDBD12 P1 ACT	DDBD165 P1 VIC	0.003
	DDBD49 P1 ACT	DDBD165 P1 VIC	0.003
	DDBD50 P1 ACT	DDBD165 P1 VIC	0.003
	DDBD58 P1 ACT	DDBD165 P1 VIC	0.003
bdM27_10_X7_874	DDBD12 P2 ACT	DDBD49 P2 ACT	0.001
	DDBD12 P2 ACT	DDBD50 P2 ACT	0.001
	DDBD12 P2 ACT	DDBD58 P2 ACT	0.001
	DDBD49 P2 ACT	DDBD58 P2 ACT	0.002
	DDBD50 P2 ACT	DDBD58 P2 ACT	0.002
	DDBD12 P2 ACT	DDBD165 P2 VIC	0.005
	DDBD49 P2 ACT	DDBD165 P2 VIC	0.004
	DDBD50 P2 ACT	DDBD165 P2 VIC	0.004
	DDBD58 P2 ACT	DDBD165 P2 VIC	0.006
of bdM27_82_X5_636	DDBD50 P4 ACT	DDBD111 P4 VIC	0.004
	DDBD364 P4 ACT	DDBD111 P4 VIC	0.004
	DDBD50 P4 ACT	DDBD117 P4 VIC	0.004
	DDBD364 P4 ACT	DDBD117 P4 VIC	0.004
	DDBD50 P4 ACT	DDBD122 P4 VIC	0.004
	DDBD364 P4 ACT	DDBD122 P4 VIC	0.004
	DDBD111 P4 VIC	DDBD145 P4 VIC	0.004
	DDBD117 P4 VIC	DDBD145 P4 VIC	0.004
	DDBD122 P4 VIC	DDBD145 P4 VIC	0.004
	DDBD50 P4 ACT	DDBD165 P4 VIC	0.009
	DDBD364 P4 ACT	DDBD165 P4 VIC	0.009
	DDBD111 P4 VIC	DDBD165 P4 VIC	0.004
	DDBD117 P4 VIC	DDBD165 P4 VIC	0.004
	DDBD122 P4 VIC	DDBD165 P4 VIC	0.004

	DDBD145 P4 VIC	DDBD165 P4 VIC	0.009
bdM27_79_X5_643	DDBD12 P5 ACT	DDBD111 P5 VIC	0.017
	DDBD49 P5 ACT	DDBD111 P5 VIC	0.017
	DDBD50 P5 ACT	DDBD111 P5 VIC	0.017
	DDBD58 P5 ACT	DDBD111 P5 VIC	0.017
	DDBD12 P5 ACT	DDBD112 P5 VIC	0.017
	DDBD49 P5 ACT	DDBD112 P5 VIC	0.017
	DDBD50 P5 ACT	DDBD112 P5 VIC	0.017
	DDBD58 P5 ACT	DDBD112 P5 VIC	0.017
	DDBD111 P5 VIC	DDBD112 P5 VIC	0.008
	DDBD12 P5 ACT	DDBD117 P5 VIC	0.013
	DDBD49 P5 ACT	DDBD117 P5 VIC	0.013
	DDBD50 P5 ACT	DDBD117 P5 VIC	0.013
	DDBD58 P5 ACT	DDBD117 P5 VIC	0.013
	DDBD111 P5 VIC	DDBD117 P5 VIC	0.004
	DDBD112 P5 VIC	DDBD117 P5 VIC	0.004
	DDBD12 P5 ACT	DDBD165 P5 VIC	0.013
	DDBD49 P5 ACT	DDBD165 P5 VIC	0.013
	DDBD50 P5 ACT	DDBD165 P5 VIC	0.013
	DDBD58 P5 ACT	DDBD165 P5 VIC	0.013
	DDBD111 P5 VIC	DDBD165 P5 VIC	0.004
	DDBD112 P5 VIC	DDBD165 P5 VIC	0.004
bdM27_69_X9_658	DDBD58 P6 ACT	DDBD111 P6 VIC	0.009
	DDBD58 P6 ACT	DDBD117 P6 VIC	0.009
	DDBD58 P6 ACT	DDBD122 P6 VIC	0.009
	DDBD111 P6 VIC	DDBD145 P6 ACT	0.009
	DDBD117 P6 VIC	DDBD145 P6 ACT	0.009
	DDBD122 P6 VIC	DDBD145 P6 ACT	0.009
	DDBD58 P6 ACT	DDBD165 P6 VIC	0.009
	DDBD111 P6 VIC	DDBD165 P6 VIC	0.006
	DDBD117 P6 VIC	DDBD165 P6 VIC	0.006
	DDBD122 P6 VIC	DDBD165 P6 VIC	0.006
	DDBD145 P6 ACT	DDBD165 P6 VIC	0.009
	DDBD111 P6 VIC	DDBD364 P6 ACT	0.009
	DDBD117 P6 VIC	DDBD364 P6 ACT	0.009
	DDBD122 P6 VIC	DDBD364 P6 ACT	0.009
	DDBD165 P6 VIC	DDBD364 P6 ACT	0.009
	DDBD111 P6 VIC	DDBDG P6 ACT	0.009
	DDBD117 P6 VIC	DDBDG P6 ACT	0.009
	DDBD122 P6 VIC	DDBDG P6 ACT	0.009
	DDBD165 P6 VIC	DDBDG P6 ACT	0.009

Table S2. BLAST results for Y-specific contigs queried against representative reptile genomes, indicating hits for the protein coding gene *UBEH2* from contig bdM27_23_X5_798; no other significant hits were found. We also searched against representative bird genomes (see main text) but recovered no significant hits. Here we report only matches with an E-value less than 10^{-5}.

Genome assembly	Scaffold	Score	E-value	Identities	Gaps	Strand
Pseudonaja textilis EBS10Xv2-PRI	ULFR01000216.1	83.5	9.00E-14	48/50 (96%)	0/50 (0%)	Plus/Minus
Pogona vitticeps pvi1.1	CEMB01027918.1	95.4	3.00E-17	51/52 (98%)	0/52 (0%)	Plus/Plus
Notechis scutatus TS10Xv2	ULFQ01011403.1	83.5	9.00E-14	48/50 (96%)	0/50 (0%)	Plus/Minus
Sphenodon punctatus ASM311381v1	QEPC01000343.1	95.4	6.00E-17	51/52 (98%)	0/52 (0%)	Plus/Plus
Varanus komodoensis ASM479886v1	SJPD01000001.1	107	6.00E-21	54/54 (100%)	0/54 (0%)	Plus/Minus
Anolis carolinensis AnoCar2.0	GL343708.1	91.4	4.00E-16	55/58 (95%)	0/58 (0%)	Plus/Plus
Crocodylus porosus CroPor_comp1	MDVP01000050.1	85.5	3.00E-14	56/60 (93%)	1/60 (2%)	Plus/Minus

Table S3. Hits to known repeats in the Dfam database [61]. Each row indicates a hit of a *B. duperreyi* contig to an entry in the Dfam database, showing the repeat model's ID, name, match score and E-value and the position of the hit in that model.

B. duperreyi **contig name**	**model accession**	**model name**	**bit score**	**e-value**	**model start**	**model end**	**strand**	**alignment start**	**alignment end**	**envelope start**	**envelope end**
bdM27_82_X5_636	DF0006275	DIRS-1e_Amnio	34.9	9.80E-11	4421	4871	+	23	481	10	507
bdM27_79_X5_643	DF0004176	RLTR6-int	92.6	2.40E-28	1409	1969	+	6	560	1	581
bdM27_69_X9_658	DF0004173	RLTR4_MM-int	44.2	9.40E-14	1200	1693	+	161	657	140	658

Table S4. Specimen data, sex, locality and measurements for the *Bassiana duperreyi* specimens used in this study. Specimen numbers refer to the University of Canberra Wildlife Tissue Collection (Genebank UC <Aus>).

Field No	Specimen No.	Sex	Sex Basis	Maturity	Population	Latitude	Longitude	SVL (mm)	Specimen status
65DM	AA064225	M	Hemipenal extrusion (testis)	Adult	Piccadilly Circus	-35.35775	148.8051444	64	Focal animal
64DM	AA064226	F	Hemipenal extrusion (ovary)	Adult	Piccadilly Circus	-35.35775	148.8051444	71	Focal animal
DDBD_3	AA064426	M	Hemipenal extrusion	Adult	Piccadilly Circus	-35.36165833	148.8034583	59	Validation animal
DDBD_4	AA064427	M	Hemipenal extrusion	Adult	Piccadilly Circus	-35.36165833	148.8034583	70	Validation animal
DDBD_5	AA064428	M	Hemipenal extrusion	Adult	Piccadilly Circus	-35.36165833	148.8034583	59	Validation animal
DDBD_7	AA064430	M	Hemipenal extrusion	Adult	Piccadilly Circus	-35.36165833	148.8034583	64	Validation animal
DDBD_8	AA064431	M	Hemipenal extrusion	Adult	Piccadilly Circus	-35.36165833	148.8034583	67	Validation animal
DDBD_9	AA064432	M	Hemipenal extrusion	Adult	Piccadilly Circus	-35.36165833	148.8034583	64	Validation animal
DDBD_12	AA064435	M	Hemipenal extrusion	Adult	Piccadilly Circus	-35.36165833	148.8034583	64	Validation animal
DDBD_13	AA064436	M	Hemipenal extrusion	Adult	Piccadilly Circus	-35.35775	148.8051444	74	Validation animal
DDBD_14	AA064437	M	Hemipenal extrusion	Adult	Piccadilly Circus	-35.35775	148.8051444	60	Validation animal
DDBD_16	AA064439	M	Hemipenal extrusion	Adult	Piccadilly Circus	-35.35775	148.8051444	64	Validation animal
DDBD_18	AA064441	M	Hemipenal extrusion	Adult	Piccadilly Circus	-35.35775	148.8051444	73	Validation animal
DDBD_19	AA064442	M	Hemipenal extrusion	Adult	Piccadilly Circus	-35.35775	148.8051444	74	Validation animal
DDBD_21	AA064444	M	Hemipenal extrusion	Adult	Piccadilly Circus	-35.35775	148.8051444	77	Validation animal
DDBD_22	AA064445	M	Hemipenal extrusion	Adult	Piccadilly Circus	-35.35775	148.8051444	66	Validation animal
DDBD_26	AA064449	M	Hemipenal extrusion	Adult	Piccadilly Circus	-35.36165833	148.8034583	73	Validation animal
DDBD_28	AA064451	M	Hemipenal extrusion	Adult	Piccadilly Circus	-35.36240556	148.8023111	61	Validation animal
DDBD_29	AA064047	M	Hemipenal extrusion	Adult	Piccadilly Circus	-35.36240556	148.8023111	63	Validation animal
DDBD_31	AA064049	M	Hemipenal extrusion	Adult	Piccadilly Circus	-35.36240556	148.8023111	71	Validation animal
DDBD_32	AA064050	M	Hemipenal extrusion	Adult	Piccadilly Circus	-35.36240556	148.8023111	70	Validation animal
DDBD_33	AA064051	M	Hemipenal extrusion	Adult	Piccadilly Circus	-35.36240556	148.8023111	65	Validation animal
DDBD_42	AA064060	M	Hemipenal extrusion	Adult	Piccadilly Circus	-35.36240556	148.8023111	64	Validation animal
DDBD_43	AA064061	M	Hemipenal extrusion	Adult	Piccadilly Circus	-35.36240556	148.8023111	62	Validation animal
DDBD_44	AA064062	M	Hemipenal extrusion	Adult	Piccadilly Circus	-35.36240556	148.8023111	69	Validation animal
DDBD_45	AA064063	M	Hemipenal extrusion	Adult	Piccadilly Circus	-35.36240556	148.8023111	59	Validation animal
DDBD_10	AA064433	F	Hemipenal extrusion (Gravid)	Adult	Piccadilly Circus	-35.35775	148.8051444	60	Validation animal

DDBD_15	AA064438	F	Hemipenal extrusion (Gravid)	Adult	Piccadilly Circus	-35.35775	148.8051444	72	Validation animal
DDBD_17	AA064440	F	Hemipenal extrusion	Adult	Piccadilly Circus	-35.35775	148.8051444	58	Validation animal
DDBD_20	AA064443	F	Hemipenal extrusion	Adult	Piccadilly Circus	-35.36165833	148.8034583	66	Validation animal
DDBD_23	AA064446	F	Hemipenal extrusion	Adult	Piccadilly Circus	-35.36240556	148.8023111	69	Validation animal
DDBD_24	AA064447	F	Hemipenal extrusion (Gravid)	Adult	Piccadilly Circus	-35.36240556	148.8023111	66	Validation animal
DDBD_25	AA064448	F	Hemipenal extrusion (Gravid)	Adult	Piccadilly Circus	-35.36240556	148.8023111	66	Validation animal
DDBD_27	AA064450	F	Hemipenal extrusion	Adult	Piccadilly Circus	-35.36240556	148.8023111	76	Validation animal
DDBD_30	AA064048	F	Hemipenal extrusion	Adult	Piccadilly Circus	-35.36240556	148.8023111	62	Validation animal
DDBD_35	AA064053	F	Hemipenal extrusion	Adult	Piccadilly Circus	-35.36240556	148.8023111	66	Validation animal
DDBD_36	AA064054	F	Hemipenal extrusion (Gravid)	Adult	Piccadilly Circus	-35.36240556	148.8023111	75	Validation animal
DDBD_39	AA064057	F	Hemipenal extrusion	Adult	Piccadilly Circus	-35.36240556	148.8023111	75	Validation animal
DDBD_40	AA064058	F	Hemipenal extrusion	Adult	Piccadilly Circus	-35.36240556	148.8023111	79	Validation animal
DDBD_41	AA064059	F	Hemipenal extrusion	Adult	Piccadilly Circus	-35.36240556	148.8023111	68	Validation animal
DDBD_47	AA064065	F	Hemipenal extrusion	Adult	Piccadilly Circus	-35.35775	148.8051444	75	Validation animal
DDBD_56	AA064074	F	Hemipenal extrusion (Gravid)	Adult	Piccadilly Circus	-35.36050833	148.8005528	63	Validation animal
DDBD_57	AA064075	F	Hemipenal extrusion (Gravid)	Adult	Piccadilly Circus	-35.36050833	148.8005528	67	Validation animal
DDBD_59	AA064077	F	Hemipenal extrusion	Adult	Piccadilly Circus	-35.36050833	148.8005528	73	Validation animal
DDBD_60	AA064078	F	Hemipenal extrusion	Adult	Piccadilly Circus	-35.36050833	148.8005528	72	Validation animal
DDBD_62	AA064080	F	Hemipenal extrusion (Gravid)	Adult	Piccadilly Circus	-35.36050833	148.8005528	66	Validation animal
DDBD_100	AA064118	F	Hemipenal extrusion (Gravid)	Adult	Piccadilly Circus	-35.36050833	148.8005528	71	Validation animal
DDBD_287	AA094000	F	Hemipenal extrusion (Gravid)	Adult	Piccadilly Circus	-35.36050833	148.8005528	73	Validation animal
DDBD_288	AA094001	F	Hemipenal extrusion (Gravid)	Adult	Piccadilly Circus	-35.36050833	148.8005528	69	Validation animal
DDBD_289	AA094002	F	Hemipenal extrusion (Gravid)	Adult	Piccadilly Circus	-35.36050833	148.8005528	67	Validation animal
DDBD_111	AA064130	M	Hemipenal extrusion	Adult	Anglesea	-38.39111111	144.215	59	Validation animal
DDBD_115	AA084795	M	Hemipenal extrusion	Adult	Anglesea	-38.39111111	144.215	62	Validation animal
DDBD_116	AA084796	M	Hemipenal extrusion	Adult	Anglesea	-38.39111111	144.215	66	Validation animal
DDBD_117	AA084798	M	Hemipenal extrusion	Adult	Anglesea	-38.39111111	144.215	73	Validation animal
DDBD_120	AA084805	M	Hemipenal extrusion	Adult	Anglesea	-38.39111111	144.215	67	Validation animal
DDBD_121	AA084806	M	Hemipenal extrusion	Adult	Anglesea	-38.39111111	144.215	67	Validation animal
DDBD_122	AA084808	M	Hemipenal extrusion	Adult	Anglesea	-38.39111111	144.215	58	Validation animal

DDBD_125	AA084813	M	Hemipenal extrusion	Adult	Anglesea	-38.39111111	144.215	60	Validation animal
DDBD_126	AA084815	M	Hemipenal extrusion	Adult	Anglesea	-38.39111111	144.215	68	Validation animal
DDBD_129	AA084373	M	Hemipenal extrusion	Adult	Anglesea	-38.39111111	144.215	59	Validation animal
DDBD_110	AA064126	F	Hemipenal extrusion	Adult	Anglesea	-38.39111111	144.215	63	Validation animal
DDBD_112	AA064135	F	Hemipenal extrusion	Adult	Anglesea	-38.39111111	144.215	62	Validation animal
DDBD_113	AA064134	F	Hemipenal extrusion	Adult	Anglesea	-38.39111111	144.215	70	Validation animal
DDBD_114	AA084791	F	Hemipenal extrusion	Adult	Anglesea	-38.39111111	144.215	66	Validation animal
DDBD_118	AA084800	F	Hemipenal extrusion	Adult	Anglesea	-38.39111111	144.215	61	Validation animal
DDBD_119	AA084802	F	Hemipenal extrusion	Adult	Anglesea	-38.39111111	144.215	62	Validation animal
DDBD_123	AA084810	F	Hemipenal extrusion	Adult	Anglesea	-38.39111111	144.215	74	Validation animal
DDBD_124	AA084811	F	Hemipenal extrusion	Adult	Anglesea	-38.39111111	144.215	73	Validation animal
DDBD_128	AA084370	F	Hemipenal extrusion	Adult	Anglesea	-38.39111111	144.215	75	Validation animal
DDBD_130	AA084374	F	Hemipenal extrusion	Adult	Anglesea	-38.39111111	144.215	70	Validation animal
DDBD_49	AA064067	M	Hemipenal extrusion	Adult	Piccadilly Circus	-35.36050833	148.8005528	65	Validation animal
DDBD_50	AA064068	M	Hemipenal extrusion	Adult	Piccadilly Circus	35.36050833	148.8005528	69	Validation animal
DDBD_58	AA064076	M	Hemipenal extrusion	Adult	Piccadilly Circus	35.36050833	148.8005528	71	Validation animal
DDBD_364	AA094425	M	Hemipenal extrusion	Adult	Piccadilly Circus	35.36050833	148.8005528	64	Validation animal
DDBD_G	AA094426	M	Hemipenal extrusion	Adult	Piccadilly Circus	35.36050833	148.8005528	62	Validation animal
DDBD_165	AA080794	M	Hemipenal extrusion	Adult	Anglesea	-38.39111111	144.215	58	Validation animal

References

[1] Kimura M. A simple method for estimating evolutionary rates of base substitutions through comparative studies of nucleotide sequences. J Mol Evol. 1980;16:111-20.

[2]. Kumar S, Stecher G, Tamura K. MEGA7: molecular evolutionary genetics analysis version 7.0 for bigger datasets. Mol Biol Evol. 2016;33:1870-4.

[3]. Hasegawa M, Kishino H, Yano TA. Dating of the human-ape splitting by a molecular clock of mitochondrial DNA. J Mol Evol. 1985 Oct 1;22(2):160-74.

[4]. Jukes TH, Cantor CR. Evolution of protein molecules. Mammalian protein metabolism. 1969;3:132.

[5]. Tamura K, Nei M, Kumar S. Prospects for inferring very large phylogenies by using the neighbor-joining method. PNAS. 2004;101:11030-5.
