# Supplementary material for: Identification of Y chromosome markers in the eastern three-lined skink (Bassiana duperreyi) using in silico whole genome subtraction
Source: BMC Genomics. 2020 Sep 29;21:667. doi: 10.1186/s12864-020-07071-2 (PMC7526180; doi:10.1186/s12864-020-07071-2)

Supplemental Materials

Raw Gel Images used to construct Fig. 1

Dissanayake, D.S.B., Holleley, C.E., Hill, L., O'Meally, D., Deakin, J., and Georges, A. (2020). Identification of Y chromosome markers in the eastern three-lined skink (*Bassiana duperreyi*) using *in silico* whole genome subtraction. BMC Genomics, in press.

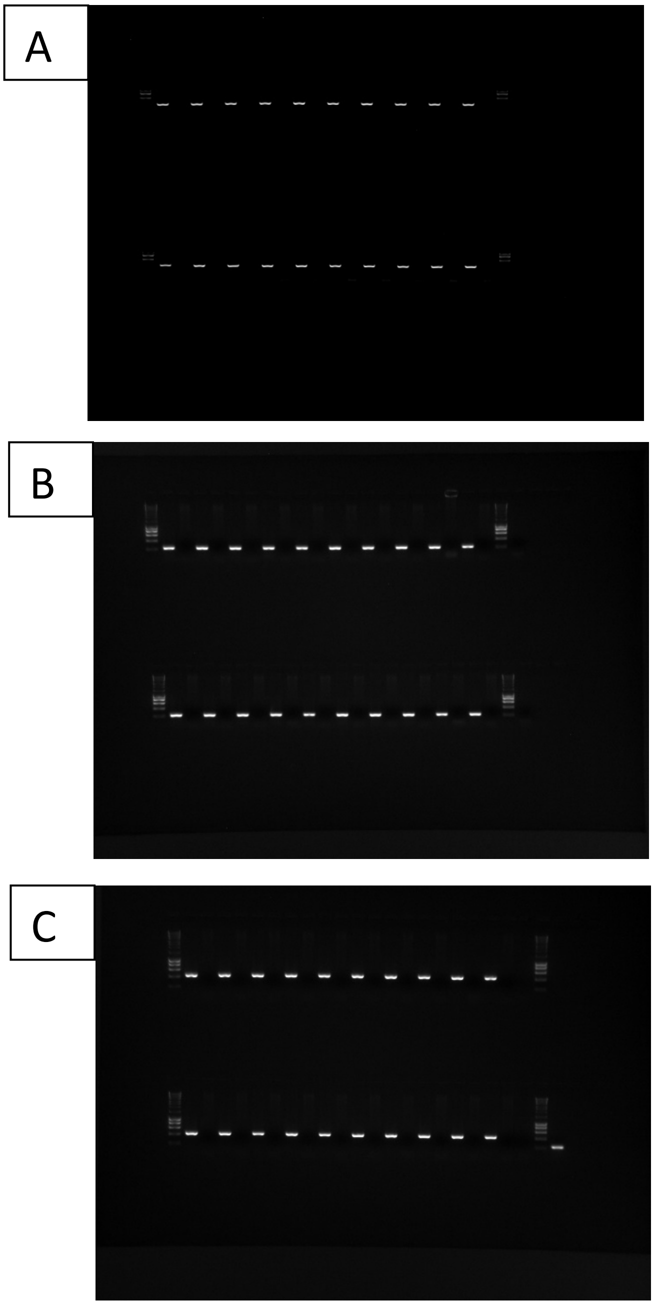

Fig S15. Raw gel images for primer sets A. bdM27_10_X7_874, B. bdM27_87_X6_628, C. bdM27_23_X5_798, D. bdM27_69_X9_658, E. bdM27_74_X11_649, F. bdM27_79_X5_643, G. bdM27_82_X5_636. Each row represents alternating Male (band) and Female (no band) individuals (n = 20) spanned by ladders. Individuals from left to right in each gel are; 1st row – DDBD_8, DDBD_23, DDBD_9, DDBD_24, DDBD_12, DDBD_25, DDBD_13, DDBD_27, DDBD_14, DDBD_30, DDBD_16, DDBD_35, DDBD_18, DDBD_36, DDBD_19, DDBD_39, DDBD_21, DDBD_40, DDBD_22, DDBD_41; 2nd row DDBD_26, DDBD_47, DDBD_28, DDBD_56, DDBD_29, DDBD_57, DDBD_31, DDBD_59, DDBD_32, DDBD_60, DDBD_33, DDBD_62, DDBD_42, DDBD_100, DDBD_43, DDBD_287, DDBD_44, DDBD_288, DDBD_45, DDBD_289. Detailed specimen list available in the Additional file 1: Table S4.

D

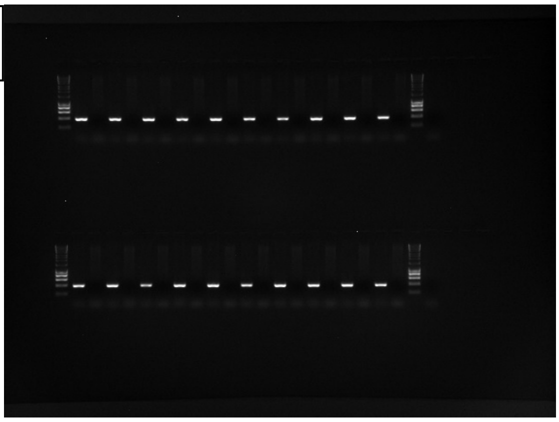

F

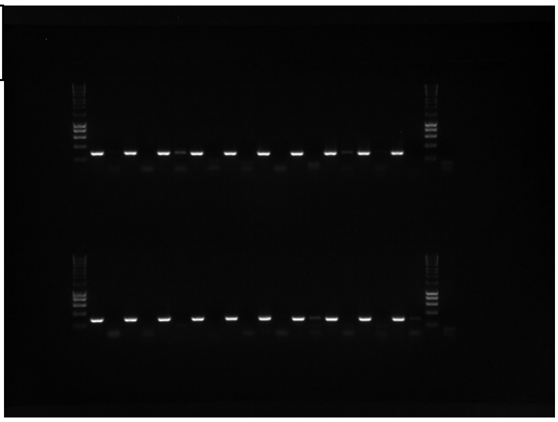

G

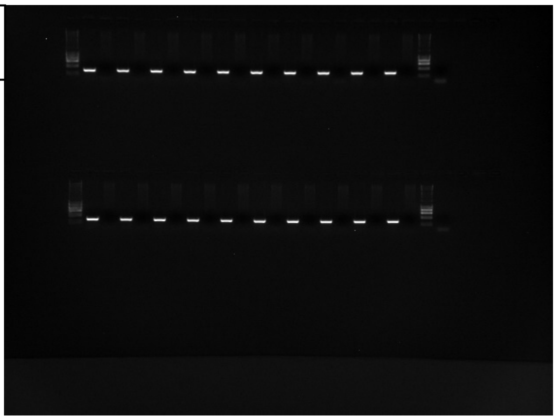

Supplement: Supplementary file 2 — Additional file 2: Original gel images to accompany Figure 1 of the manuscript. Figure S15. Raw gel images for primer sets A. bdM27_10_X7_874, B. bdM27_87_X6_628, C. bdM27_23_X5_798, D. bdM27_69_X9_658, E. bdM27_74_X11_649, F. bdM27_79_X5_643, G. bdM27_82_X5_636. Each row represents alternating Male (band) and Female (no band) individuals (n = 20) spanned by ladders. Individuals from left to right in each gel are; 1st row – DDBD_8, DDBD_23, DDBD_9, DDBD_24, DDBD_12, DDBD_25, DDBD_13, DDBD_27, DDBD_14, DDBD_30, DDBD_16, DDBD_35, DDBD_18, DDBD_36, DDBD_19, DDBD_39, DDBD_21, DDBD_40, DDBD_22, DDBD_41; 2nd row DDBD_26, DDBD_47, DDBD_28, DDBD_56, DDBD_29, DDBD_57, DDBD_31, DDBD_59, DDBD_32, DDBD_60, DDBD_33, DDBD_62, DDBD_42, DDBD_100, DDBD_43, DDBD_287, DDBD_44, DDBD_288, DDBD_45, DDBD_289. Detailed specimen list available in the Additional file 1: Table S4. [file 12864_2020_7071_MOESM2_ESM.pdf]
